# A Low-Dose CT-Based Radiomic Model to Improve Characterization and Screening Recall Intervals of Indeterminate Prevalent Pulmonary Nodules

**DOI:** 10.3390/diagnostics11091610

**Published:** 2021-09-03

**Authors:** Leonardo Rundo, Roberta Eufrasia Ledda, Christian di Noia, Evis Sala, Giancarlo Mauri, Gianluca Milanese, Nicola Sverzellati, Giovanni Apolone, Maria Carla Gilardi, Maria Cristina Messa, Isabella Castiglioni, Ugo Pastorino

**Affiliations:** 1 Department of Radiology, University of Cambridge, Cambridge CB2 0QQ, UK; es220@medschl.cam.ac.uk; 2 Cancer Research UK Cambridge Centre, University of Cambridge, Cambridge CB2 0RE, UK; 3 Unit of Radiological Sciences, Department of Medicine and Surgery (DiMeC), University of Parma, 43126 Parma, Italy; robertaeufrasia.ledda@unipr.it (R.E.L.); gianluca.milanese@unipr.it (G.M.); nicola.sverzellati@unipr.it (N.S.); 4 Fondazione IRCCS Istituto Nazionale dei Tumori di Milano, 20133 Milan, Italy; giovanni.apolone@istitutotumori.mi.it (G.A.); ugo.pastorino@istitutotumori.mi.it (U.P.); 5 Department of Physics “Giuseppe Occhialini”, University of Milano-Bicocca, 20126 Milan, Italy; c.dinoia@campus.unimib.it; 6 Department of Informatics, Systems and Communication, University of Milano-Bicocca, 20126 Milan, Italy; giancarlo.mauri@unimib.it; 7 School of Medicine and Surgery, University of Milano-Bicocca, 20126 Milan, Italy; maria.gilardi@unimib.it (M.C.G.); cristina.messa@unimib.it (M.C.M.); 8 Institute of Biomedical Imaging and Physiology, Italian National Research Council (IBFM-CNR), Segrate, 20090 Milan, Italy; 9 Fondazione Tecnomed, University of Milano-Bicocca, 20900 Monza, Italy

**Keywords:** pulmonary nodules, lung cancer screening, low-dose computed tomography, lung cancer risk stratification, radiomics, machine learning

## Abstract

Lung cancer (LC) is currently one of the main causes of cancer-related deaths worldwide. Low-dose computed tomography (LDCT) of the chest has been proven effective in secondary prevention (i.e., early detection) of LC by several trials. In this work, we investigated the potential impact of radiomics on indeterminate prevalent pulmonary nodule (PN) characterization and risk stratification in subjects undergoing LDCT-based LC screening. As a proof-of-concept for radiomic analyses, the first aim of our study was to assess whether indeterminate PNs could be automatically classified by an LDCT radiomic classifier as solid or sub-solid (first-level classification), and in particular for sub-solid lesions, as non-solid versus part-solid (second-level classification). The second aim of the study was to assess whether an LCDT radiomic classifier could automatically predict PN risk of malignancy, and thus optimize LDCT recall timing in screening programs. Model performance was evaluated using the area under the receiver operating characteristic curve (AUC), accuracy, positive predictive value, negative predictive value, sensitivity, and specificity. The experimental results showed that an LDCT radiomic machine learning classifier can achieve excellent performance for characterization of screen-detected PNs (mean AUC of 0.89 ± 0.02 and 0.80 ± 0.18 on the blinded test dataset for the first-level and second-level classifiers, respectively), providing quantitative information to support clinical management. Our study showed that a radiomic classifier could be used to optimize LDCT recall for indeterminate PNs. According to the performance of such a classifier on the blinded test dataset, within the first 6 months, 46% of the malignant PNs and 38% of the benign ones were identified, improving early detection of LC by doubling the current detection rate of malignant nodules from 23% to 46% at a low cost of false positives. In conclusion, we showed the high potential of LDCT-based radiomics for improving the characterization and optimizing screening recall intervals of indeterminate PNs.

## 1. Introduction

Lung cancer (LC) accounts for up to 18.4% of all cancer-related deaths worldwide [1,2]. Up to 70% of patients suffer from advanced disease—either locally advanced (stage IIIC) or metastatic (stage IV)—at the time of diagnosis, limiting curative options [3,4]. LC secondary prevention (i.e., early detection) by means of low-dose computed tomography (LDCT) has been proven effective by several LC screening (LCS) trials, showing a 20–30% mortality reduction in high-risk subjects [1,5,6,7].

Pulmonary nodule (PN) is the most common imaging presentation of LC and the most frequent LDCT finding in LCS (detected in up to 70% of cases) [8,9]. Currently, their management mostly relies on size and density, with the former being the key parameter for predicting LC risk and assigning LDCT-based outcome categories [10] and for which volumetry is the current recommended metric [11,12,13]. Based on density, PNs are classified into solid and sub-solid (SN and SSN, respectively), with SSN further differentiated into two subcategories: pure non-solid (or ground-glass) nodules (NSNs) and part-solid nodules (PSNs), which contain both non-solid and solid components [14,15]. The measurement of solid components, typically the largest diameter of the solid component, is currently used by radiologists to support the discrimination of negative from positive PN at LDCT. The vast majority of screen-detected PN, however, are benign or malignancies that would have not affected subjects’ life expectancy due to low tumor aggressiveness or concurrent diseases [16,17,18]. Despite the efforts to improve the accuracy of LC risk stratification, the rate of such indeterminate PNs, which carry a 5 to 65% probability of being malignant, remains high in LCS, requiring additional investigations with a subsequent increase of both economic and human costs (i.e., psychological burden) [19]. Moreover, there are currently no adequate strategies to recall patients with indeterminate PN to carry out LDCT controls based on personalized risks. This means that, for some of them, if not recalled within the first six months from the baseline LDCT, the diagnosis may occur after one year from the indeterminate PN finding.

Radiomics is an emerging translational field of research aiming at converting medical images into mineable data by extracting a large number of quantitative features that would be either overlooked or undetectable with the naked eye [20]. Radiomics has been proven useful in risk stratification, screening, diagnosis, and prognosis of several diseases, mostly in oncology [21,22,23,24], including the early diagnosis of small PNs [25].

In this work, we aimed at investigating the potential impact of radiomics on the management of patients with indeterminate PN in a National LCS trial, called bioMILD. The first aim of our study was to assess whether indeterminate prevalent PNs could be automatically classified as SN or SSN based on a LDCT radiomic classifier, allowing for a more objective and reproducible PN characterization in LCS. The second aim was to assess whether a radiomic classifier could predict the risk of malignancy of indeterminate prevalent PN, and, thus, improve risk stratification.

## 2. Materials and Methods

### 2.1. The bioMILD Trial

The bioMILD trial (*clinicaltrials.gov* ID: NCT02247453), a single-center LCS trial performed at the “Istituto Nazionale dei Tumori di Milano”, Milan, Italy, prospectively enrolled between January 2013 and March 2016, 4119 subjects with a median age of 60 years, median cigarette pack-years of 42, current smokers 79% and females 39%. At the end of March 2019, a total of 11,012 LDCTs were performed, with an overall compliance at the 3-year LDCT of 93% and a median follow-up of 4.2 years. This study was approved by the local Institutional Review Board “Comitato Etico Indipendente—Fondazione IRCCS Istituto Nazionale dei Tumori di Milano” (Prot. INT 21/11) approved on 28 April 2011.

#### 2.1.1. Imaging Acquisition and Interpretation

For each subject of the bioMILD trial, LDCT scans were performed on a second-generation dual-source CT scanner (Somatom Definition Flash; Siemens Medical Solutions; Forchheim, Germany). LDCT images were acquired with the subject in the supine position during end inspiration breath-hold. CT acquisition parameters were as follows: tube voltage 120 kVp, tube current 30 mAs, collimation 0.625 mm, pitch 1.2, rotation time 0.5 s. Images were reconstructed with the following parameters: slice thickness 1 mm; increment 0.7 mm; medium-sharp kernel (B50f); lung window setting (window width 1600 Hounsfield Units, HU, window level −600 HU).

Images were visually assessed by two radiologists—the first reader used a computer-aided detection (CAD) system (MM.Oncology, Syngo.via; Siemens Healthcare; Erlangen, Germany), whereas the second reader adopted Maximum Intensity Projection (MIP) tools. The first reader was responsible for the creation of the report, which was evaluated—after image analysis—by the second reader. In case of disagreement (e.g., identification of nodules not included in the report by the first reader), the second radiologist was allowed to edit the report. These edits were then discussed by the two readers to reach a final decision, in consensus, on the LDCT outcome. The integration of tools for the detection of pulmonary nodules (e.g., CAD) within the workflow strengthened the opinions in a highly reproducible manner.

The LDCT outcome was assigned based on a visually-assessed type of nodule (i.e., SN, PSN, or NSN) and size—assessed by volumetric measurements performed by the CAD software and checked for correctness by the reading radiologist for SNs and PSNs, while size for NSN was assessed by measuring the diameters by electronic calipers, thus resulting in:

- Negative LDCT (LDCT-): no PN detected, nodule with fat or benign pattern of calcifications, SN < 113 mm^3^ or NSN < 5 mm;
- Indeterminate LDCT (LDCT Ind): SN 113–260 mm^3^, PSN with solid component < 5 mm or NSN > 5 mm;
- Positive LDCT (LDCT+): SN > 260 mm^3^, PSN with solid component > 5 mm.

#### 2.1.2. Quantitative Analysis and Radiomic Feature Measurement

Baseline LDCT Ind were retrieved from the local Picture Archiving and Communication System (PACS) and, following data anonymization, uploaded into a dedicated open-source software (3D Slicer 4.10.0, www.slicer.org (accessed on 1 September 2021)).

PNs were semi-automatically delineated every two slices through manually drawn regions of interest (ROIs) by a radiologist with one year experience in thoracic imaging, and the remaining slices were interpolated accordingly. A dedicated algorithmic tool was then used to calculate a volume of interest (VOI), including the whole lesions. In case of inaccurate segmentation, the operator was allowed to modify VOI boundaries.

Radiomic features were extracted using a segmentation software built-in function, named SlicerRadiomics, which integrates the tool PyRadiomics (v2.2.0) [26] that is aimed at measuring standardized radiomic features [27,28]. Six classes of features were obtained: (1) first-order intensity histogram statistics, (2) Gray Level Co-occurrence Matrix features (GLCM) [29,30,31], (3) Gray Level Run Length Matrix (GLRLM) [32], (4) Gray Level Size Zone Matrix (GLSZM) [33], (5) Gray Level Dependence Matrix (GLDM) [34], and (6) Neighboring Gray Tone Difference Matrix (NGTDM) [35]. A fixed bin width of 25 was used and no resampling was applied. All the radiomic features are listed in Table S5.

#### 2.1.3. Dataset Composition

The whole dataset included 703 PNs. Such a dataset was divided into 2 datasets: a discovery and a blinded data set, through a 66–34% split hold-out approach.

The discovery dataset included two datasets:

1. 544 PNs (dataset I), classified into SNs (324, 59.6%) and SSNs (220, 40.4%), of which 55 (25%) were PSNs and 165 (75%) NSNs—based on LDCT density measured by the readers as previously described (see Section 2.1.1) and used as reference standard I [36];
2. 326 PNs (dataset II), sent to biopsy and then classified into malignant (32, 9.8%) and benign (294, 90.2%), based on histopathological features (reference standard II).

The blinded test dataset included two datasets:

1. 159 PNs (dataset III), classified into SNs (81, 50.9%) and SSNs (78, 49.1%)—further classified into PSNs (29, 37.2%) and NSNs (49, 62.8%);
2. 104 PNs (dataset IV), sent to biopsy and then classified as malignant (13, 12.5%) and benign (91, 87.5%).

Datasets I and II were used to train and validate radiomic automatic classifiers supervised by the respective reference standards, as described in the following sections, while datasets III and IV were used to test such classifiers in a blinded way, that is, without knowing the reference standard, thus mimicking the real-world clinical condition. For this purpose, the research group of “Istituto Nazionale dei Tumori di Milano” sent the reference standards of datasets III and IV to the research group of the University of Milan-Bicocca, only at the end of the model development, after classification of PNs from this blinded test dataset has been provided.

### 2.2. Radiomic Analyses

The analyses were aimed at:

- Aim 1 (PN characterization): developing a SN vs. SSN radiomic automatic classifier. For this purpose, datasets I and III were used. Moreover, a sub-classification of SSN into NSN vs. PSN was performed by a second-level radiomic classifier.
- Aim 2 (PN risk): developing a malignant vs. benign nodule radiomic automatic classifier. For this purpose, datasets II and IV were used.

**Figure 1:**
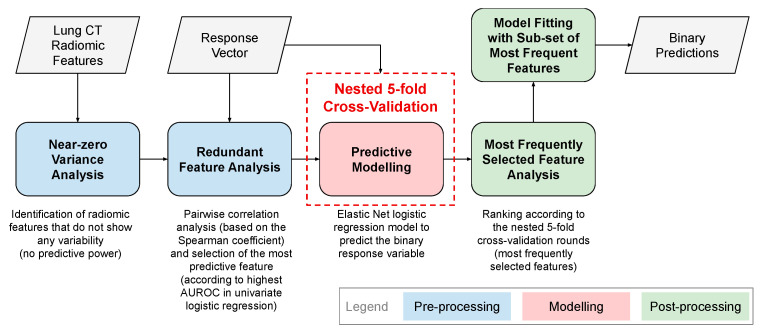
Overall workflow for the development of LDCT radiomic automatic classifiers for PNs.

For both aims, the overall data analysis workflow is shown in Figure 1 and includes the measurement of LDCT radiomic features from PNs, a data pre-processing procedure, the development of the predictive model for binary classification (according to the two aforementioned aims) based on selected radiomic features, and a post-processing procedure.

All data analyses were performed using the MatLab^®^ R2019b (64-bit version) environment (MathWorks, Natick, MA, USA). The evaluation metrics used were the area under the receiver operating characteristic curve (AUC) and classification accuracy, along with positive predictive value (PPV) and negative predictive value (NPV), sensitivity, and specificity.

The characteristics of the proposed radiomics study according to the reporting guidelines provided by the Image Biomarker Standardization Initiative (IBSI) [28] are provided in Supplementary Table S6.

#### 2.2.1. Pre-Processing of Radiomic Features

Two pre-processing operations were applied on the extracted radiomic features [37].

##### Near-Zero Variance Analysis

Near-zero variance analysis was aimed at removing the radiomic features that did not convey information content. This operation considers a cutoff for the ratio of the most common value to the second most common value and a cutoff for the percentage of distinct values out of the number of total samples. We used the default values 95/5 and 10 for the respective cutoffs.

##### Redundant Feature Analysis

The goal of this step was to remove highly correlated radiomic features for reducing the redundancy among the features. We used the Spearman correlation coefficient *ρ_S_* for pairwise feature comparison. In the case of |*ρ_S_*| ≥ 0.90, the feature with the highest predictive power was selected. This choice was performed by a univariate logistic regression for predicting the binary lesion characterization and removing the feature that achieved the lowest AUC.

#### 2.2.2. Cross-Validation of Radiomic Classifiers on Discovery Datasets

The two radiomic classifiers were implemented using the Elastic Net regularization for logistic regression. Output (response) variables were, for Aim 1 (PN characterization), the binary nodule density (PN into SN vs. SSN, and SSN into PSN vs. NSN based on solid component diameter, SN and PSN being the positive and SSN and NSN being the negative classes, respectively), and for Aim 2 (PN risk), the histological nodule diagnosis (malignant vs. benign, malignant being the positive and benign being the negative class, respectively) [38].

Elastic Net uses a mixture between ℓ_1_ and ℓ_2_ regularization. The ℓ_1_ regularization—also known as Least Absolute Shrinkage and Selection Operator (LASSO) [39,40]—reduces the coefficients of certain features to zero, thus reducing the number of variables in a sparse model. The ℓ_2_ penalty term—also called ridge regression [41]—constrains the magnitude of the feature coefficients so that a model is not dominated by any single feature. As a hyper-parameter tuning, we considered *α* ∈ {0.10, 0.25, 0.50, 0.75, 0.90, 1.0}, α being the weight for ℓ_1_ and ℓ_2_ penalties, also known as the mixing parameter.

The radiomic classifiers were fitted on the respective discovery cohorts using nested 5-fold cross-validation (CV). Specifically, the classifier for Aim 1 (PN characterization at the two levels) was trained in the inner CV loop with dataset I, whereas the classifier for Aim 2 (PN risk) was trained in the inner CV loop with dataset III. A nested 5-fold CV was chosen since it allows for model training when model hyper-parameters need to be optimized [42]. The hyper-parameter selection (*λ* in the case of the Elastic Net regularization) by means of non-nested CV could indeed yield a biased model, leading to over-optimistic performance [43].

The best performing models were selected according to the maximum classification accuracy in terms of AUC. To estimate the performance of the models, the fitting was repeated 50 times with different random permutations of the discovery dataset. We averaged the performance of the models across such independent repetitions.

During the inner CV loop, the optimal operating point of the ROC curve was estimated by using the slope *s* according to Equation (1):

(1) $$s=\frac{\mathrm{Cos}t(P|N)-\mathrm{Cos}t(N|N)}{\mathrm{Cos}t(N|P)-\mathrm{Cos}t(P|P)}\times \frac{N}{P},$$

where Cost(N|P) and Cost(P|N) are the costs of misclassifying a positive class as a negative class and a negative class as a positive class, respectively; *P* and *N* denote the total numbers in the positive and negative class, respectively. Therefore, the optimal operating point was defined by the intersection of the straight line with slope *s* from the upper left corner of the ROC axes (False Positive Ratio = 0, True Positive Ratio = 1) and the ROC curve [44].

The majority voting of the ensemble based on the single classifier prediction was used to employ the optimized decision thresholds.

#### 2.2.3. Post-Processing of Radiomic Features: Radiomic Predictors

Relying upon the achieved radiomic classifier results, we selected the most relevant radiomic features in terms of their occurrences, that is, choosing the features most frequently found as predictors by the classifiers. Therefore, Elastic Net was re-fitted on this reduced subset of radiomic features using the same nested *k*-fold CV scheme and settings. Elastic Net was then applied to the discovery datasets (also in terms of data partitioning) as previously done for all the radiomic features.

#### 2.2.4. Integration and Comparisons of Radiomic Predictors with Clinical Features and Semantic LDCT Features

Along with the most relevant radiomic features (best radiomic predictors), the following features were considered and integrated to re-train and test Elastic Net on the discovery datasets (as described in Section 3):

- Best solidity radiomic features: best radiomic predictors of Aim 1, as an objective measure of solidity of each PN;
- Clinical features: body mass index (BMI), forced expiratory volume in 1 second (FEV1), and C-reactive protein (CRP);
- Semantic LDCT features: emphysema extent, type (centrilobular, paraseptal, or panlobular) and location (lobes involved); anterior descending, circumflex and right coronary artery calcifications (categorical values); and bronchial wall thickening (dichotomous variable).

#### 2.2.5. Testing Radiomic Classifiers on Blinded Test Dataset

The radiomic classifiers (Aim 1: PN characterization and Aim 2: PN risk) fitted in 5-fold cross-validation on the discovery dataset (dataset I and III, for Aims 1 and 2, respectively) were tested on the blinded held-out datasets (dataset II and IV, for Aims 1 and 2, respectively). As previously stated, in such a way, we assessed the performance on unseen data that was kept blinded until the final evaluation and independent from the design choices during the discovery phase, simulating a real-world clinical use of the developed radiomic classifiers.

#### 2.2.6. Re-Training Classifier with Feature Class Imbalance Correction

In order to manage the problem of imbalanced classes during the training of the radiomic classifier for Aim 2 (~0.1:0.9 for malignant vs. benign PNs), we exploited different strategies to over-sample the minority class (malignant PNs) and to under-sample the majority class (benign PNs).

##### Minority Class Over-Sampling

Minority class over-sampling aims to properly manage the class imbalance in the original datasets by artificially generating synthetic samples according to the actual data distributions.

The Synthetic Minority Over-Sampling Technique (SMOTE) algorithm [45] generates an arbitrary number of synthetic minority examples to shift the classifier learning bias towards the minority class. The minority class is over-sampled by taking each minority class sample and introducing synthetic examples along the line segments joining any/all of the *k*_NN_ minority class nearest neighbors. Among SMOTE modifications, the Borderline-SMOTE algorithm [46] uses the same over-sampling technique, but it over-samples only the borderline instances of a minority class rather than considering all the instances of the class. The Safe-Level-SMOTE algorithm [47] assigns, for each positive instance, its safe-level prior to generating synthetic instances. Each synthetic instance is positioned closer to the largest safe-level so that all synthetic instances are generated only in safe regions. An alternative method is the Adaptive Synthetic (ADASYN) sampling approach [48] which is based on the idea of adaptively generating minority data samples according to their distributions: more synthetic data are generated for minority class samples that are harder to learn compared to those minority samples that are easier to learn.

We applied all these synthetic over-sampling methods to the minority class during the re-training of the radiomic classifier for Aim 2 (PN risk).

To assess the data distribution of the real and synthetic samples, we exploited the Principal Component Analysis (PCA) [49] and t-Distributed Stochastic Neighbor Embedding (t-SNE) methods [50] to obtain a 2D representation. PCA is the most-used dimensionality reduction method, which reduces the dimensions of the input feature space by finding linear combinations of the original variables that show the highest standard deviation. PCA can highly reduce the dimensions of the data without losing relevant information when the linear correlations of the data are dominant. t-SNE is a method for non-linear dimensionality reduction: it is typically used for data visualization, but can also be used to reduce the feature space, as well as for clustering. Compared to PCA, t-SNE is not a linear algebra technique, but it is based on a probabilistic framework. With more details, the divergence between two distributions is minimized: a distribution that measures pairwise similarities of the input objects and a distribution that measures pairwise similarities of the corresponding low-dimensional points in the embedding.

##### Majority Class Under-Sampling

Stratified sampling for the majority class was obtained by using different under-sampling factors (i.e., multiples of the minority class size). In particular, we sub-sampled the majority class by obtaining 32, 62, 128, 256 samples.

To deal with the dependence on the initial sub-sampling from the whole majority class samples, 30 different random sub-sampling configurations were considered. Then, the re-training procedure was performed during the 5-fold cross-validation process, randomly for 30 times. For each sub-sampling configuration, the training procedure during the 5-fold cross-validation was repeated 50 times and the performance metrics were consequently averaged. Then, the sampling configuration with the highest mean AUC was selected to be consistent with the other tests.

#### 2.2.7. Impact of Radiomic Classifiers on PN Characterization and Screening Recall Intervals

The impact of the first LDCT radiomic classifier developed for Aim 1 (PN characterization) was assessed by comparing the classifier performance against its reference standard, the LDCT nodule density (in terms of HU) on both discovery and blinded test datasets, considering the clinical needs for an objective and reproducible density lesion characterization. This comparison allowed for the demonstration of the proof-of-concept at the basis of the radiomic hypothesis assumption in our classifiers, that is, that radiomic features are able to capture the intra- and inter- lesion heterogeneity in an effective way.

The impact of the second LCDT radiomic classifier developed for Aim 2 (PN risk) was assessed by measuring the rates of subjects of both discovery and blinded test datasets recalled for a second LDCT scan, that is, the procedure recommended for monitoring indeterminate PNs in a screening program. More specifically, according to the bioMILD screening protocol, subjects with indeterminate PNs are recalled to a second LCDT scan in three classes of follow-up groups temporally distributed as follows: (i) within 0–6 months, (ii) within 12–24 months, and (iii) within 24–36 months. We computed the rates of subjects recalled in the three groups without the use of our radiomic classifier developed for PN risk. We compared these rates with the respective diagnosis of malignant or benign PNs, performed at biopsy before the recall. Then, we computed the same rates, simulating the use of our radiomic classifier as decision support to recall subjects at risk of malignancy for their second LDCT scan within the first 6 months, whereas the others (at a lower risk according to our radiomic model) were within 12 months. We finally compared the two recall rates obtained without or with the support of our radiomic model and derived the value of our classifier for all the subjects both in the discovery and blinded test datasets.

## 3. Results

### 3.1. Pre-Processing of Radiomic Features on Discovery Datasets

The near-zero variance analyses, applied to the initial set of 107 radiomic features characterizing each indeterminate prevalent PN on the discovery dataset, led to 96 and 103 informative features for the classifiers developed for Aim 1 (first-level/second-level PN characterization, respectively), while 104 informative features were found for the classification task of Aim 2 (PN risk). Univariate logistic regression for removing redundant features led to 31 and 23 informative, non-redundant features in classifiers for Aim 1 (first-level/second-level PN characterization, respectively), whereas 32 informative, non-redundant features were identified for Aim 2 (PN risk), respectively.

### 3.2. Cross-Validation of Radiomic Classifiers on Discovery Datasets

The results of the hyper-parameter optimization process in the Elastic Net for the mixing parameter α are shown in Figure S1; even though the performance was generally robust against the hyper-parameter variations, the best configuration was provided by α = 0.75.

**Figure 2:**
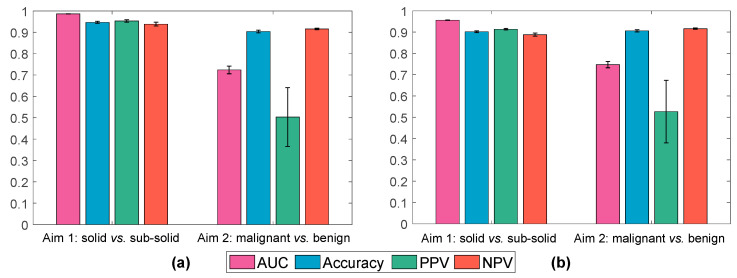
Classification results achieved by the radiomic classifiers for Aim 1 (PN characterization) and Aim 2 (PN risk) based on the Elastic Net on the discovery dataset by using (**a**) informative, non-redundant radiomic features, and (**b**) only the most frequently selected radiomic features. The bar graph and error bars denote the average value and the variability across 50 repetitions, respectively.

Figure 2a shows the results achieved by the two radiomic classifiers (Aim 1 and Aim 2) using the informative, non-redundant radiomic features on the discovery set.

The radiomic classifier built on 31 informative, non-redundant features for the first-level classification of Aim 1 (PN characterization in terms of SN vs. SSN) achieved a mean AUC, Accuracy, PPV, and NPV of 0.986 ± 0.001, 0.947 ± 0.005, 0.953 ± 0.006, and 0.939 ± 0.008, respectively. For the second-level classification of Aim 1 (SSN characterization in terms of NSN vs. PSN) using 23 informative, non-redundant features, the achieved mean AUC, Accuracy, PPV, and NPV were 0.963 ± 0.004, 0.913 ± 0.010, 0.848 ± 0.026, and 0.939 ± 0.010, respectively. These results showed the excellent ability of our radiomic system in the fine-gained automatic characterization of the density of indeterminate PNs.

The radiomic classifier developed using 32 informative and non-redundant radiomic features for Aim 2 (PN risk) achieved a mean AUC, Accuracy, PPV, and NPV of 0.727 ± 0.017, 0.904 ± 0.006, 0.515 ± 0.141, and 0.916 ± 0.003, respectively, for SN vs. SSN. These results showed the excellent ability of our radiomic system in predicting the negative class but a limitation in predicting the PNs, as expected due to the imbalanced ratio between positive and negative samples (~0.1:0.9 for malignant vs. benign PNs).

### 3.3. Post-Processing of Radiomic Features: Radiomic Predictors

Considering the 250 trained model instances, we analyzed the most frequently selected radiomic features in the discovery cohort for both Aim 1 (PN characterization) and Aim 2 (PN risk), with the purpose to select a limited number of radiomic predictors (see Figure S2a,b for Aim 1 and Aim 2, respectively).

For Aim 1 (first-level classifier), the most frequently selected features (more than 100 times) were:

- Shape: Sphericity;
- NGTDM: Contrast;
- GLSZM: Small Area High Gray Level Emphasis;
- GLRLM: Gray Level Variance.

For Aim 2, the most frequently selected features (more than 150 times) were:

- Shape: Maximum 2D Diameter Slice, Least Axis Length;
- GLCM: Correlation, Cluster Shade;
- GLSZM: Size Zone Non-Uniformity Normalized, Size Zone Non-Uniformity.

For an intuitive interpretation, such radiomic features were graphically represented by the boxplots in Figures S3 and S4 for Aim 1 and Aim 2, respectively. According to the occurrences shown in these histograms, the cutoff values (100 and 150 times, respectively) were experimentally selected to define the corresponding radiomic signatures. In particular, this process resulted in two distinct signatures composed of four and six features for Aim 1 (PN characterization) and Aim 2 (PN risk), respectively.

For statistical validation, a non-parametric Wilcoxon sum test (Mann–Whitney U test) was performed for each feature by subdividing the samples into the two classes (significance level set to 0.05). The *p*-values were adjusted using the Bonferroni–Holm method for multiple comparison tests.

Figure S3 revealed that the features appear to be significantly different, between the two sub-distributions, for all the four features (*p* < 0.0001). The distributions of the features appear visibly different in SN and SSN. From Figure S4, only one feature (GLSZM Size Zone Non-Uniformity Normalized) was found not statistically significant, while GLSZM Size Zone Non-Uniformity Normalized achieved a *p* < 0.001 and the other three features showed a *p* < 0.01. The feature distributions appear to be different in the malignant and benign nodules; however, the ranges explored in the different cases appear to overlap, at least in part.

Figure 2a shows the results achieved on the most frequently selected radiomic features.

While the Aim 1 results on the most frequently selected features (Figure 2b) were slightly lower—yet excellent for the first-level (second-level) classification in terms of AUC, accuracy, PPV, and NPV 0.956 ± 0.001 (0.967 ± 0.004), 0.902 ± 0.004 (0.915 ± 0.010), 0.913 ± 0.003 (0.866 ± 0.025), and 0.888 ± 0.007 (0.935 ± 0.008), respectively—than those on the larger informative, non-redundant features subset (Figure 2a), only a slight increase in both AUC (0.747 ± 0.015) and PPV (0.527 ± 0.147) can be observed on Aim 2, due to the problem of imbalanced classes. However, the NPV for this task is excellent (0.916 ± 0.003).

### 3.4. Integration of Radiomic Predictors with Semantic LDCT Features and Clinical Features and Comparison

Table 1 shows the performance of the radiomic classifier for Aim 2 (PN risk) obtained using the best radiomic predictors of such a classifier alone (see the previous section) compared with the performance of the same classifier re-trained on the best radiomic predictors of Aim 2 in combination with the best radiomic predictors of Aim 1 (solidity), clinical and semantic LDCT features. The performance of a classifier trained only on clinical features are also reported in Table 1, for comparison. All analyses were performed on the discovery dataset (dataset III).

As shown in Table 1, the best classifier for Aim 2 is the one based on the best radiomic predictors (AUC = 0.747 ± 0.015, Accuracy = 0.906 ± 0.005, PPV = 0.527 ± 0.147, NPV = 0.916 ± 0.003), while no additional benefits emerge from the combination with the other factors—i.e., the best radiomic predictors of Aim 1 (solidity), clinical, and semantic LDCT features.

### 3.5. Testing Radiomic Classifiers on Blinded Test Dataset

Table 2 shows the performance achieved by testing the radiomic classifiers on the blinded cohort for Aim 1 (PN characterization, dataset III), at the two levels, and Aim 2 (PN risk, dataset IV).

While Aim 1 showed excellent results in the blinded test dataset in all the evaluation metrics and at both levels (the mean values for the first and second levels were: AUC = 0.887 and 0.800, accuracy = 0.870 and 0.844, PPV = 0.830 and 0.988, NPV = 0.926 and 0.801, sensitivity = 0.938 and 0.580, specificity = 0.800 and 0.991, respectively), Aim 2 showed relatively lower sensitivity and PPV, resulting in a lower overall AUC (0.564). As already found during the discovery phase, this result is expected due to the highly imbalanced data distribution (~0.1:0.9 for malignant vs. benign PNs) and needs to be ameliorated.

### 3.6. Re-Training Classifier with Feature Class Imbalance Correction

#### 3.6.1. Minority Class Over-Sampling

Table S1 shows the classification results achieved on the discovery set by the classifier for Aim 2 with the various minority class over-sampling configurations. According to Table S1, Borderline-SMOTE generally achieved the best performance among the investigated approaches.

Table 3 shows, for the discovery dataset of Aim 2 (dataset II), that the best configuration is Borderline-SMOTE with 256 generated synthetic samples, since it achieved the highest sensitivity and PPV (0.67 and 0.83, respectively). Accuracy, specificity, and NPV values exhibited a similar trend for all four approaches, and tend to degrade as the number of generated synthetic samples increases. In all methods, *K*_NN_ = 5 has been used for the evaluation of the nearest neighbors [51].

#### 3.6.2. Majority Class under-Sampling

Table S2 shows the classification results achieved on the discovery set by the classifier for Aim 2 with the various majority class under-sampling configurations. The best configuration was obtained by a sub-sampling with 96 samples; however, sensitivity and PPV were lower than those obtained with Borderline-SMOTE with 256 samples.

Table 4 shows, for the blinded test dataset of Aim 2 (dataset II), the classifier performance for the various minority class over-sampling methods, confirming that the best configuration is Borderline-SMOTE with 256 generated synthetic samples since, also in this case, it achieved the highest sensitivity and PPV (0.46 and 0.15, respectively). Comparing these results with the sensitivity and PPV of the same classifier without minority class over-sampling, we should note an improvement of a factor of ~5× at cost of an increase of false positive of a factor of ~2×.

Table S4 reveals that all the majority class under-sampling configurations did not boost the obtained sensitivity consistently with results obtained for the discovery cohort.

### 3.7. Impact of Radiomic Classifiers on PN Characterization and Screening Recall Intervals

As shown in Section 3.2, the performance of the classifier developed for Aim 1 (first-level classification for PN characterization) on the discovery cohort (dataset I), was 0.96 and 0.90 in terms of AUC and accuracy, respectively, with a predictive value of 0.91 and 0.89 for SNs and SSNs, respectively. Similarly, the classifier developed for Aim 1 (second-level classification for PN characterization) on the discovery cohort (dataset I), was 0.97 and 0.92 in terms of AUC and accuracy, respectively, with a predictive value of 0.87 and 0.94 for NSNs and PSNs, respectively. As shown in Section 3.4, on the blinded cohort (dataset III), the first-level classifier obtained 0.89 AUC and 0.87 accuracy, with a predictive value of 0.83 and 0.93 for SNs and SSNs, respectively. The second-level classifier obtained 0.80 AUC and 0.84 accuracy, with a predictive value of 0.99 and 0.80 for NSNs and PSNs, respectively. These results demonstrate that radiomic features can easily capture the tissue density heterogeneity of indeterminate PNs at LDCT. More impactful for the subjects, such a classifier could be effectively used in clinical settings for automatically classifying the density of indeterminate PNs, thus providing an automatic objective lesion characterization to radiologists. This task is quite simple for experienced radiologists for characterization of PN into SN vs. SSN when based on the diameter measure of the solid component in the PN, but it is complex for classification of SSN into NSN and PSN, this last information being the most important added value of our first radiomic classifier developed for Aim 1 at the second-level.

We discuss, here, the result on the potential clinical impact of using our second radiomic classifier developed for Aim 2 (PN risk) in predicting malignant vs. benign PNs, thus supporting physicians in selecting the right recall interval for subjects for a second LDCT study.

**Figure 3:**
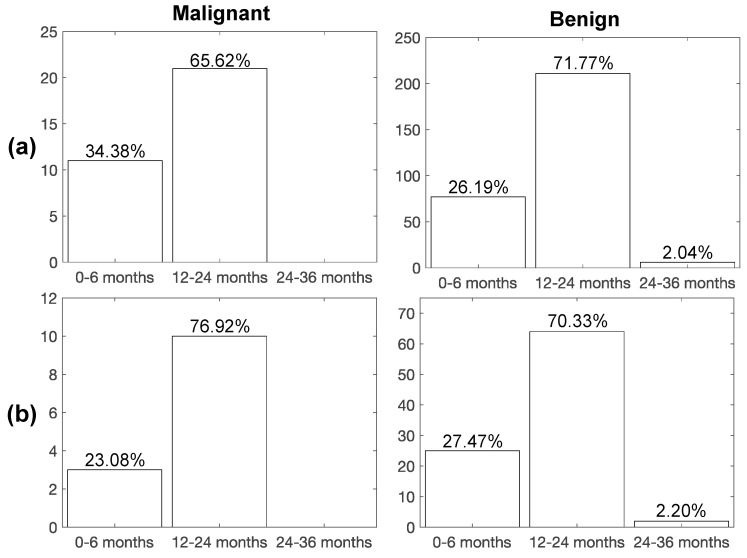
Percentage of LDCT second scan recalls for subjects with malignant and benign PN on the (**a**) discovery and (**b**) blinded test datasets, according to the reference standard (biopsy). Time interval for the second LDCT examination after LDCT baseline: (i) 0–6 months, (ii) 12–24 months, (iii) 24–36 months.

Figure 3 shows the rates of subjects of the discovery cohort (dataset II) recalled during the bioMILD screening program by physicians, without the support of our radiomic classifier, to be studied by a second LDCT scan (LCDT recall) with the purpose to monitor the subjects’ PN. The rates are shown temporally distributed into the three classes of follow-up: (i) 0–6 months, (ii) 12–24 months, and (iii) 24–36 months. The diagnosis of malignant or benign PN, performed at biopsy before the recall, is also shown in Figure 3 in order to discuss the accuracy of the recall plan.

**Figure 4:**
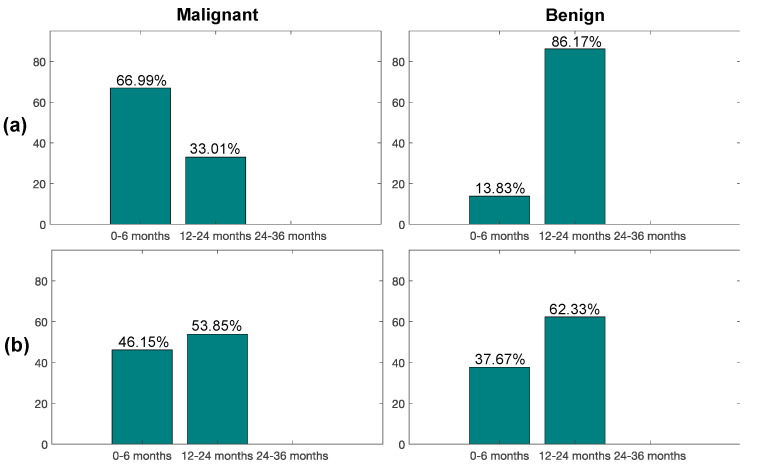
Percentage of LDCT second scan recalls for subjects with malignant and benign PN on the (**a**) discovery and (**b**) blinded test datasets, according to the radiomics-based classifier. Time interval for the second LDCT examination after LDCT baseline: (i) 0–6 months, (ii) 12–24 months, (iii) 24–36 months.

As we can observe from Figure 3a, only 34% of subjects from the total with malignant nodules were recalled in the bioMILD screening trial within 6 months from the LDCT baseline study, while 26% of subjects with benign nodules entered in this first recall window. Instead, using our second radiomic classifier (Figure 4a), most of the malignant nodules (67%) and only 14% benign ones would have been recalled within 6 months, whereas the remaining ones would have been recalled within a year (33% malignant, 86% benign), thus improving early detection of LC (improving of a factor of ~2× the portion of malignant nodules from 34% to 67%) and also reducing the number of false positives.

For the blinded cohort (dataset IV), as shown in Figure 3b, only 23% of malignant nodules were recalled in the bioMILD screening trial within 6 months from the LDCT baseline study. Instead, with the support of our radiomic classifier developed for Aim 2 (PN risk) (Figure 4b), according to the tested performance in the blinded test dataset, 46% of malignant cases and 38% benign cases would have been recalled within 6 months from the baseline LDCT round, thus improving the early detection of LC by doubling the portion of malignant nodules from 23% to 46% at a low cost of false positives.

Importantly, such a radiomic classifier could allow screenees with LC to be recalled significantly earlier than the current screening interval (*p* = 0.0079). Table 5 shows, indeed, the months between the baseline LDCT scan and LC diagnosis for both the subjects recalled at either 0–6 months or 12–24 months during the bioMILD screening program (without the use of our classifier) (18 ± 23.00 and 17 ± 23.25 in the discovery and blinded cohort, respectively) and months of the diagnosis for the subjects that would be recalled according to our radiomic classifier (44 ± 22.00 and 27 ± 18.00 in the discovery and blinded cohort, respectively). As a matter of fact, we might have identified LC patients earlier in both discovery and blinded test sets and this could have a great impact on the clinical outcome of these patients, considering the rapid progression of LC.

## 4. Discussion

During a screening program of LC, risk category assignment and subsequent management of screen-detected PN currently rely on size and density of PNs at baseline LDCT [10], with shape being also taken into account to assess the risk of malignancy. There is, however, a substantial need to improve risk stratification for indeterminate PNs, which represent the most challenging diagnostic category, requiring further evaluation and thus, leading to anxiety, increased healthcare costs, and potentially unnecessary invasive procedures [52,53].

Our study is the first one, to the best of our knowledge, in which radiomics and machine learning are applied for providing a tool that can potentially improve the management of prevalent indeterminate PNs in a lung cancer screening trial.

Huang et al. in [54] defined a radiomic signature as an independent biomarker for the estimation of disease-free survival in patients with early-stage non-small cell lung cancer. Authors demonstrated well that the combination of the radiomic signature with a traditional staging system and other clinical-pathologic risk factors performed better in estimating disease-free survival in such patients, as compared to the traditional staging system and clinical-pathologic factors. The radiomic signature was generated by using the LASSO method, combined to a Cox regression model. Further validation of the radiomic signature as an independent biomarker was performed by using a multivariate Cox regression. This approach is similar to that used in our study, which was based on LASSO with Elastic Net.

In [55], a radiomic model was developed to improve LDCT-based classification of PNs. The prediction model was constructed by using a support vector machine (SVM) classifier coupled with LASSO. A 10-fold CV was used to evaluate the accuracy of a hybrid SVM-LASSO model. The best model achieved an accuracy of 84.6%, which was 12.4% higher than that for Lung-RADS, and the AUC was 0.89. Choi et al., however, did not consider only indeterminate PNs, as in our study; thus, the reported performance could be masked by a simpler classification task obtained, including determined positive and negative PNs that have clearly different size and solid component. Specifically, negative LDCT are: no nodule detected, nodule with fat or benign pattern of calcifications, SN < 113 mm^3^ or NSN < 5 mm; positive LDCT are: SN > 260 mm^3^, PSN with solid component > 5 mm.

Recently in [56], by using the publicly available data and LDCT images from the National Lung Screening Trial (NLST), authors evaluated radiomic features describing size, shape, volume, and textural characteristics from both the intratumoral and the peritumoral region. After the first process of feature extraction, stable and reproducible radiomic features were significantly associated with overall survival (OS), achieving an AUC of 0.88 at 2-year OS. The work in [57] aimed at evaluating radiomic classifiers for early identification of malignant PNs. A first model was based on artificial neural networks, while a second one was based on a SVM classifier coupled with LASSO. The AUC performance of the models was >0.89 in the training set and >0.82 in the external validation set for all the investigated scenarios, outperforming the clinical standard (AUC of 0.76). Results showed a good accuracy of the investigated models in distinguishing benign from malignant PNs but, also in this case, no selection on prevalent indeterminate PN was performed and no specific results on the classifier performance were provided.

As proof-of-concept for radiomic analyses, we showed that radiomics can accurately discriminate solid and subsolid lesions detected as prevalent indeterminate PNs, not only in discriminating solid vs. sub-solid lesions (AUC > 98%) but, more importantly, in the more complex task of characterizing a sub-solid lesion into either part-solid or non-solid (AUC > 89%). Our first classification systems developed for these purposes (PN characterization at two levels) could thus potentially assist radiologists, providing an objective assessment of screen-detected indeterminate PNs.

Regarding our second radiomic classifiers developed to predict malignancy of indeterminate PNs, the main achievements of our model would not be a saving in terms of number of LDCT scans to be performed (since its sensitivity and specificity performance is not sufficiently adequate to avoid the recall of PN-detected subjects within 24 months from the baseline round), but to allow for a personalized recall system. Indeed, from the analysis of the study cohorts of the considered screening trails, it was observed that the majority of malignant PNs were not recalled within 6 months from the baseline LDCT (only 34% and 23% of the malignant PNs for the discovery and blinded test datasets, respectively), but within 12 months. Based on our predictive radiomic model, a higher proportion of malignant PNs, namely 67% for the discovery dataset and 38% for the blinded one, would have been recalled within 6 months from the baseline LDCT, potentially avoiding late treatment, with a positive impact on both human health and economic costs.

Furthermore, a significant proportion of subjects recalled within 6 months could have been recalled within 12 or even 24 to 36 months, with substantial advantages for screening programs, for whom the psychological burden would have been more bearable, and for the health system economic costs [58]. Interestingly, with such a radiomic model, we might have identified LC patients earlier, recalling them for a second LDCT around after 0–6 months rather than after 12–24 months.

This study, however, has a few limitations. First, the retrospective design might be prone to some confounding factors, including the selection of some patients. Indeed, the deployment of radiomics to “real-world” scenarios, where imaging data are not controlled by specific trial protocols but retrieved from the current clinical practice, is still challenging to implement [59]. In particular, in compliance with this very recent study, a nested cross-validation was adopted, along with hyper-parameter tuning, to avoid over-optimistic results despite our single-center study. However, we exploited the homogeneity intrinsic in our dataset (collected in a clinical trial) that enabled careful processing and assessment of the extracted radiomic features; this experimental procedure increases the result reliability, as well as the model generalization on a fully blinded test cohort. Second, the proposed classifiers have not been validated on an external independent cohort yet since a second LDCT screening dataset with indeterminate PNs is not currently available from an external center or in public databases. However, external test cohorts would be required to assess the generalization abilities of the developed radiomic classifiers.

To conclude, we showed the high potential of LDCT-based radiomics classifiers for optimizing the image characterization and screening intervals of second LDCT tests in subjects with indeterminate PNs detected at baseline LDCT. Future work will be aimed at integrating multiple data streams, such as microRNA signatures or other genetic features, to better characterize the screened subjects from a geno-phenotype profiling [60], along with the use of molecular imaging [61,62].

## Figures and Tables

**Table 1:** Results on Aim 2 were achieved by incrementally integrating the best radiomic predictors of Aim 2 with the best radiomic predictors of Aim 1 (solidity), available clinical and semantic LDCT radiological features. Values in boldface denote the best configuration.

Features	AUC	Accuracy	PPV	NPV
**Radiomic**	**0.747 ± 0.015**	**0.906 ± 0.005**	**0.527 ± 0.147**	**0.916 ± 0.003**
Best radiomic predictors of Aim 2 + best radiomic predictors of Aim 1 (solidity)	0.739 ± 0.014	0.905 ± 0.005	0.522 ± 0.137	0.915 ± 0.003
Best radiomic predictors of Aim 2 + best radiomic predictors of Aim 1 (solidity) + clinical + semantic LDCT	0.742 ± 0.021	0.898 ± 0.006	0.423 ± 0.120	0.913 ± 0.003
Clinical + semantic LDCT	0.521 ± 0.036	0.893 ± 0.006	0.039 ± 0.076	0.902 ± 0.002

**Table 2:** Performance achieved by the radiomic classifiers fitted (nested 5-fold CV) on the blinded test set for Aim 1 (PN characterization) and Aim 2 (PN risk). Metrics are expressed as average ± standard deviation.

	AUC	Accuracy	PPV	NPV	Sensitivity	Specificity
**Aim 1 first-level:** SN vs. SSN	0.887 ± 0.006	0.870 ± 0.010	0.830 ± 0.014	0.926 ± 0.002	0.938 ± 0.001	0.800 ± 0.020
**Aim 1 second-level:** NSN vs. PSN	0.800 ± 0.178	0.844 ± 0.005	0.988 ± 0.002	0.801 ± 0.005	0.580 ± 0.0134	0.991 ± 0.001
**Aim 2:** benign vs. malignant	0.564 ± 0.007	0.869 ± 0.005	0.367 ± 0.079	0.886 ± 0.004	0.102 ± 0.036	0.978 ± 0.001

**Table 3:** Results for the benign vs. malignant primary nodule classification (Aim 2) achieved by the nested 5-fold CV on the discovery set for the Borderline-SMOTE over-sampling method. The metrics are expressed as average ± standard deviation. The rows in boldface denote the best performing configuration according to sensitivity and PPV.

# Samples	AUC	Accuracy	PPV	NPV	Sensitivity	Specificity
32	0.768 ± 0.008	0.826 ± 0.007	0.568 ± 0.061	0.857 ± 0.007	0.292 ± 0.043	0.946 ± 0.009
64	0.788 ± 0.004	0.809 ± 0.008	0.669 ± 0.033	0.839 ± 0.008	0.432 ± 0.038	0.928 ± 0.012
128	0.826 ± 0.004	0.794 ± 0.007	0.741 ± 0.013	0.820 ± 0.008	0.644 ± 0.021	0.875 ± 0.009
192	0.795 ± 0.002	0.783 ± 0.006	0.806 ± 0.010	0.772 ± 0.005	0.657 ± 0.009	0.878 ± 0.009
**256**	**0.817 ± 0.003**	**0.766 ± 0.006**	**0.830 ± 0.009**	**0.726 ± 0.004**	**0.670 ± 0.006**	**0.862 ± 0.009**
295	0.802 ± 0.003	0.749 ± 0.005	0.840 ± 0.009	0.688 ± 0.004	0.648 ± 0.006	0.861 ± 0.010

**Table 4:** Results for the benign vs. malignant primary nodule classification (Aim 2) achieved by the classifiers fitted (in nested 5-fold CV) on the blinded test set for the Borderline-SMOTE method. The metrics are expressed as average ± standard deviation. The rows in boldface denote the best performing configuration according to sensitivity and PPV.

# Samples	AUC	Accuracy	PPV	NPV	Sensitivity	Specificity
32	0.602 ± 0.012	0.838 ± 0.019	0.317 ± 0.067	0.894 ± 0.002	0.229 ± 0.011	0.925 ± 0.021
64	0.591 ± 0.016	0.805 ± 0.018	0.229 ± 0.028	0.890 ± 0.002	0.231 ± 2.24 × 10^−16^	0.887 ± 0.021
128	0.571 ± 0.009	0.697 ± 0.018	0.163 ± 0.011	0.889 ± 0.004	0.346 ± 0.039	0.747 ± 0.024
192	0.574 ± 0.008	0.643 ± 0.008	0.165 ± 0.006	0.896 ± 0.003	0.457 ± 0.019	0.670 ± 0.010
**256**	**0.556 ± 0.007**	**0.603 ± 0.010**	**0.149 ± 0.004**	**0.890 ± 0.002**	**0.462 ± 4.49 × 10^−16^**	**0.623 ± 0.011**
295	0.548 ± 0.009	0.584 ± 0.009	0.142 ± 0.003	0.887 ± 0.002	0.462 ± 4.49 × 10^−16^	0.601 ± 0.011

**Table 5:** Months of LC diagnosis from baseline LDCT scan in subjects recalled at 0–6 months. Month distributions for the LDCT recalls, as well as for the LC predictions, yielded by the proposed radiomic classifier, are expressed as median ± interquartile range values. Month distributions of the detection of LC with the proposed radiomic classifier are compared against the month distributions of the reference standard LDCT recalls after 0–6 months using the Wilcoxon rank sum test (with a significance level of 0.05). The *p*-values were adjusted using the Bonferroni–Holm method for multiple comparison tests.

Dataset	Months of LC Diagnosis from Baseline LDCT in Subjects Recalled at 0–6 Months without the Use of the Radiomic Classifier for Aim 2 (PN Risk)	Months of LC Diagnosis from Baseline LDCT in Subjects to Be Recalled at 0–6 Months with the Use of the Radiomic Classifier for Aim 2 (PN Risk)
Discovery	18 ± 23.00	44 ± 22.00; *p* = 0.0079
Blinded test	17 ± 23.25	27 ± 18.00; *p* = 0.5000

## Data Availability

Data cannot be made publicly available due to ethical restrictions.

## Supplementary Materials

The following are available online at https://www.mdpi.com/article/10.3390/diagnostics11091610/s1, Figure S1: Hyper-parameter optimisation of the radiomic models in terms of the mixing parameter α (Elastic Net ℓ_1_/ℓ_2_ regularization parameter), Figure S2: Most frequently selected radiomic feature analysis of the Elastic Net models, Figure S3: Boxplots of the four most frequently selected radiomic features for Aim 1 solid vs. sub-solid nodule classification on the discovery dataset, Figure S4: Boxplots of the six most frequently selected radiomic features for Aim 2 malignant vs. benign primary nodule classification on the discovery dataset, Figure S5. Dimensionality reduction to assess the data distribution for the synthetic samples generated by Borderline-SMOTE on the discovery set of primary malignant nodules (Aim 1) for all the 107 radiomic features, as well as for the most frequently selected ones, Figure S6. Distributions of the synthetic samples generated by Borderline-SMOTE compared against the actual minority class samples (i.e. malignant primary nodules) included in the discovery set, Table S1. Results for the benign vs. malignant primary nodule classification (Aim 2) achieved by the nested 5-fold CV on the discovery set for each minority class over-sampling method under consideration, Table S2. Results for the benign vs. malignant primary nodule classification (Aim 2) achieved by the nested 5-fold CV on the discovery set for each majority class under-sampling method under consideration. The metrics are expressed as average ± standard deviation, Table S3. Results for the benign vs. malignant primary nodule classification (Aim 2) achieved by the fitted models (in nested 5-fold CV) on the blinded test set for each minority class over-sampling method under consideration, Table S4. Results for the benign vs. malignant primary nodule classification (Aim 2) achieved by the fitted models (in nested 5-fold CV) on the blinded test set for each minority class under-sampling method under consideration, Table S5. Radiomic features extracted from the VOIs in this study, Table S6. Characteristics of the proposed radiomics study according to the reporting guidelines provided by the Image Biomarker Standardization Initiative (IBSI) [Chapter 4].

## References

[1] De Koning H.J., van der Aalst C.M., de Jong P.A., Scholten E.T., Nackaerts K., Heuvelmans M.A., Lammers J.-W.J., Weenink C., Yousaf-Khan U., Horeweg N. (2020). Reduced Lung-Cancer Mortality with Volume CT Screening in a Randomized Trial. N. Engl. J. Med..

[2] Aberle D.R., Berg C.D., Black W.C., Church T.R., Fagerstrom R.M., Galen B., Gareen I.F., Gatsonis C., Goldin J., National Lung Screening Trial Research Team (2011). The National Lung Screening Trial: Overview and Study Design. Radiology.

[3] Goldstraw P., Chansky K., Crowley J., Rami-Porta R., Asamura H., Eberhardt W.E.E., Nicholson A.G., Groome P., Mitchell A., Bolejack V. (2016). The IASLC Lung Cancer Staging Project: Proposals for Revision of the TNM Stage Groupings in the Forthcoming (Eighth) Edition of the TNM Classification for Lung Cancer. J. Thorac. Oncol..

[4] Saul E.E., Guerra R.B., Saul M.E., da Silva L.L., Aleixo G.F.P., Matuda R.M.K., Lopes G. (2020). The Challenges of Implementing Low-Dose Computed Tomography for Lung Cancer Screening in Low- and Middle-Income Countries. Nat. Cancer.

[5] Aberle D.R., Adams A.M., Berg C.D., Black W.C., Clapp J.D., Fagerstrom R.M., Gareen I.F., Gatsonis C., Marcus P.M., National Lung Screening Trial Research Team (2011). Reduced Lung-Cancer Mortality with Low-Dose Computed Tomographic Screening. N. Engl. J. Med..

[6] Pastorino U., Silva M., Sestini S., Sabia F., Boeri M., Cantarutti A., Sverzellati N., Sozzi G., Corrao G., Marchianò A. (2019). Prolonged Lung Cancer Screening Reduced 10-Year Mortality in the MILD Trial: New Confirmation of Lung Cancer Screening Efficacy. Ann. Oncol..

[7] Hunger T., Wanka-Pail E., Brix G., Griebel J. (2021). Lung Cancer Screening with Low-Dose CT in Smokers: A Systematic Review and Meta-Analysis. Diagnostics.

[8] Tammemagi M.C., Schmidt H., Martel S., McWilliams A., Goffin J.R., Johnston M.R., Nicholas G., Tremblay A., Bhatia R., Liu G. (2017). Participant Selection for Lung Cancer Screening by Risk Modelling (the Pan-Canadian Early Detection of Lung Cancer [PanCan] Study): A Single-Arm, Prospective Study. Lancet Oncol..

[9] Swensen S.J., Jett J.R., Sloan J.A., Midthun D.E., Hartman T.E., Sykes A.-M., Aughenbaugh G.L., Zink F.E., Hillman S.L., Noetzel G.R. (2002). Screening for Lung Cancer with Low-Dose Spiral Computed Tomography. Am. J. Respir. Crit. Care Med..

[10] Lung–RADS® Version 1.1, Assessment Categories (Release date: 2019). https://www.acr.org/-/media/ACR/Files/RADS/Lung-RADS/LungRADSAssessmentCategoriesv1-1.pdf?la=en.

[11] Gierada D.S., Rydzak C.E., Zei M., Rhea L. (2020). Improved Interobserver Agreement on Lung-RADS Classification of Solid Nodules Using Semiautomated CT Volumetry. Radiology.

[12] De Margerie-Mellon C., Gill R.R., Monteiro Filho A.C., Heidinger B.H., Onken A., VanderLaan P.A., Bankier A.A. (2020). Growth Assessment of Pulmonary Adenocarcinomas Manifesting as Subsolid Nodules on CT: Comparison of Diameter-Based and Volume Measurements. Acad. Radiol..

[13] Borghesi A., Michelini S., Scrimieri A., Golemi S., Maroldi R. (2019). Solid indeterminate pulmonary nodules of less than 300 mm^3^: Application of different volume doubling time cut-offs in clinical practice. Diagnostics.

[14] Godoy M.C.B., Naidich D.P. (2009). Subsolid Pulmonary Nodules and the Spectrum of Peripheral Adenocarcinomas of the Lung: Recommended Interim Guidelines for Assessment and Management. Radiology.

[15] Borghesi A., Farina D., Michelini S., Ferrari M., Benetti D., Fisogni S., Tironi A., Maroldi R. (2016). Pulmonary adenocarcinomas presenting as ground-glass opacities on multidetector CT: Three-dimensional computer-assisted analysis of growth pattern and doubling time. Diagn. Interv. Radiol..

[16] Esserman L.J., Thompson I.M., Reid B. (2013). Overdiagnosis and Overtreatment in Cancer: An Opportunity for Improvement. JAMA.

[17] Carter J.L., Coletti R.J., Harris R.P. (2015). Quantifying and Monitoring Overdiagnosis in Cancer Screening: A Systematic Review of Methods. BMJ.

[18] Bach P.B. (2008). Overdiagnosis in Lung Cancer: Different Perspectives, Definitions, Implications. Thorax.

[19] Wu G.X., Raz D.J., Brown L., Sun V. (2016). Psychological Burden Associated With Lung Cancer Screening: A Systematic Review. Clin. Lung Cancer.

[20] Gillies R.J., Kinahan P.E., Hricak H. (2016). Radiomics: Images Are More than Pictures, They Are Data. Radiology.

[21] Castiglioni I., Rundo L., Codari M., Di Leo G., Salvatore C., Interlenghi M., Gallivanone F., Cozzi A., D’Amico N.C., Sardanelli F. (2021). AI Applications to Medical Images: From Machine Learning to Deep Learning. Phys. Med..

[22] Ninatti G., Kirienko M., Neri E., Sollini M., Chiti A. (2020). Imaging-Based Prediction of Molecular Therapy Targets in NSCLC by Radiogenomics and AI Approaches: A Systematic Review. Diagnostics.

[23] Mazzaschi G., Milanese G., Pagano P., Madeddu D., Gnetti L., Trentini F., Falco A., Frati C., Lorusso B., Lagrasta C. (2020). Integrated CT Imaging and Tissue Immune Features Disclose a Radio-Immune Signature with High Prognostic Impact on Surgically Resected NSCLC. Lung Cancer.

[24] Antunovic L., Gallivanone F., Sollini M., Sagoma A., Invento A., Manfrinato G., Kirienko M., Tinterri C., Chiti A. (2017). [18F]FDG PET/CT features for the molecular characterization of primary breast tumors. Eur. J. Nucl. Med. Mol. Imaging.

[25] Huang P., Park S., Yan R., Lee J., Chu L.C., Lin C.T., Hussien A., Rathmell J., Thomas B., Chen C. (2018). Added Value of Computer-Aided CT Image Features for Early Lung Cancer Diagnosis with Small Pulmonary Nodules: A Matched Case-Control Study. Radiology.

[26] Fedorov A., Beichel R., Kalpathy-Cramer J., Finet J., Fillion-Robin J.-C., Pujol S., Bauer C., Jennings D., Fennessy F., Sonka M. (2012). 3D Slicer as an Image Computing Platform for the Quantitative Imaging Network. Magn. Reson. Imaging.

[27] Zwanenburg A., Vallières M., Abdalah M.A., Aerts H.J.W.L., Andrearczyk V., Apte A., Ashrafinia S., Bakas S., Beukinga R.J., Boellaard R. (2020). The Image Biomarker Standardization Initiative: Standardized Quantitative Radiomics for High-Throughput Image-Based Phenotyping. Radiology.

[28] Zwanenburg A., Leger S., Vallières M., Löck S. (2016). Image biomarker standardisation initiative. arXiv.

[29] Haralick R.M., Shanmugam K., Dinstein I. (1973). Textural Features for Image Classification. IEEE Trans. Syst. Man Cybern..

[30] Haralick R.M. (1979). Statistical and Structural Approaches to Texture. Proc. IEEE.

[31] Rundo L., Tangherloni A., Galimberti S., Cazzaniga P., Woitek R., Sala E. (2019). HaraliCU: GPU-powered Haralick feature extraction on medical images exploiting the full dynamics of gray-scale levels. Proceedings of the International Conference on Parallel Computing Technologies (PaCT) 2019.

[32] Galloway M.M. (1975). Texture Analysis Using Gray Level Run Lengths. Comput. Graph. Image Process..

[33] Thibault G., Angulo J., Meyer F. (2014). Advanced Statistical Matrices for Texture Characterization: Application to Cell Classification. IEEE Trans. Biomed. Eng..

[34] Sun C., Wee W.G. (1982). Neighboring Gray Level Dependence Matrix for Texture Classification. Comput. Graph. Image Process..

[35] Amadasun M., King R. (1989). Textural Features Corresponding to Textural Properties. IEEE Trans. Syst. Man Cybern..

[36] Revel M.-P., Mannes I., Benzakoun J., Guinet C., Léger T., Grenier P., Lupo A., Fournel L., Chassagnon G., Bommart S. (2018). Subsolid Lung Nodule Classification: A CT Criterion for Improving Interobserver Agreement. Radiology.

[37] Papanikolaou N., Matos C., Koh D.M. (2020). How to Develop a Meaningful Radiomic Signature for Clinical Use in Oncologic Patients. Cancer Imaging.

[38] Zou H., Hastie T. (2005). Regularization and Variable Selection via the Elastic Net. J. R. Stat. Soc. B.

[39] Tibshirani R. (2011). Regression Shrinkage and Selection via the Lasso: A Retrospective. R. Stat. Soc. B.

[40] Gill A.B., Rundo L., Wan J.C.M., Lau D., Zawaideh J.P., Woitek R., Zaccagna F., Beer L., Gale D., Sala E. (2020). Correlating Radiomic Features of Heterogeneity on CT with Circulating Tumor DNA in Metastatic Melanoma. Cancers.

[41] Hoerl A.E., Kennard R.W. (2000). Ridge Regression: Biased Estimation for Nonorthogonal Problems. Technometrics.

[42] Parvandeh S., Yeh H.-W., Paulus M.P., McKinney B.A. (2020). Consensus Features Nested Cross-Validation. Bioinformatics.

[43] Cawley G.C. (2012). Over-Fitting in Model Selection and Its Avoidance. Advances in Intelligent Data Analysis XI (IDA 2012).

[44] Briggs W.M., Zaretzki R. (2008). The Skill Plot: A Graphical Technique for Evaluating Continuous Diagnostic Tests. Biometrics.

[45] Chawla N.V., Bowyer K.W., Hall L.O., Kegelmeyer W.P. (2002). SMOTE: Synthetic Minority Over-Sampling Technique. J. Artif. Intell. Res..

[46] Han H., Wang W.-Y., Mao B.-H. (2005). Borderline-SMOTE: A New Over-Sampling Method in Imbalanced Data Sets Learning. Advances in Intelligent Computing (ICIC 2005).

[47] Bunkhumpornpat C., Sinapiromsaran K., Lursinsap C. (2009). Safe-Level-SMOTE: Safe-Level-Synthetic Minority Over-Sampling TEchnique for Handling the Class Imbalanced Problem. Advances in Knowledge Discovery and Data Mining.

[48] He H., Bai Y., Garcia E.A., Li S. ADASYN: Adaptive Synthetic Sampling Approach for Imbalanced Learning. Proceedings of the 2008 IEEE International Joint Conference on Neural Networks (IEEE World Congress on Computational Intelligence) 2008.

[49] Hotelling H. (1933). Analysis of a Complex of Statistical Variables into Principal Components. J. Educ. Psychol..

[50] Van der Maaten L., Hinton G. (2008). Visualizing Data Using t-SNE. J. Mach. Learn. Res..

[51] Ramentol E., Vluymans S., Verbiest N., Caballero Y., Bello R., Cornelis C., Herrera F. (2015). IFROWANN: Imbalanced Fuzzy-Rough Ordered Weighted Average Nearest Neighbor Classification. IEEE Trans. Fuzzy Syst..

[52] Lokhandwala T., Bittoni M.A., Dann R.A., D’Souza A.O., Johnson M., Nagy R.J., Lanman R.B., Merritt R.E., Carbone D.P. (2017). Costs of Diagnostic Assessment for Lung Cancer: A Medicare Claims Analysis. Clin. Lung Cancer.

[53] Freiman M.R., Clark J.A., Slatore C.G., Gould M.K., Woloshin S., Schwartz L.M., Wiener R.S. (2016). Patients’ Knowledge, Beliefs, and Distress Associated with Detection and Evaluation of Incidental Pulmonary Nodules for Cancer: Results from a Multicenter Survey. J. Thorac. Oncol..

[54] Huang Y., Liu Z., He L., Chen X., Pan D., Ma Z., Liang C., Tian J., Liang C. (2016). Radiomics Signature: A Potential Biomarker for the Prediction of Disease-Free Survival in Early-Stage (I or II) Non-Small Cell Lung Cancer. Radiology.

[55] Choi W., Oh J.H., Riyahi S., Liu C.-J., Jiang F., Chen W., White C., Rimner A., Mechalakos J.G., Deasy J.O. (2018). Radiomics Analysis of Pulmonary Nodules in Low-Dose CT for Early Detection of Lung Cancer. Med. Phys..

[56] Pérez-Morales J., Tunali I., Stringfield O., Eschrich S.A., Balagurunathan Y., Gillies R.J., Schabath M.B. (2020). Peritumoral and Intratumoral Radiomic Features Predict Survival Outcomes among Patients Diagnosed in Lung Cancer Screening. Sci. Rep..

[57] Garau N., Paganelli C., Summers P., Choi W., Alam S., Lu W., Fanciullo C., Bellomi M., Baroni G., Rampinelli C. (2020). External Validation of Radiomics-Based Predictive Models in Low-Dose CT Screening for Early Lung Cancer Diagnosis. Med. Phys..

[58] Silva M., Milanese G., Sestini S., Sabia F., Jacobs C., van Ginneken B., Prokop M., Schaefer-Prokop C.M., Marchianò A., Sverzellati N. (2021). Lung Cancer Screening by Nodule Volume in Lung-RADS v1.1: Negative Baseline CT Yields Potential for Increased Screening Interval. Eur. Radiol..

[59] Doran S.J., Kumar S., Orton M., d’Arcy J., Kwaks F., O’Flynn E., Ahmed Z., Downey K., Dowsett M., Turner N. (2021). “Real-world” radiomics from multi-vendor MRI: An original retrospective study on the prediction of nodal status and disease survival in breast cancer, as an exemplar to promote discussion of the wider issues. Cancer Imaging.

[60] Boeri M., Verri C., Conte D., Roz L., Modena P., Facchinetti F., Calabrò E., Croce C.M., Pastorino U., Sozzi G. (2011). MicroRNA Signatures in Tissues and Plasma Predict Development and Prognosis of Computed Tomography Detected Lung Cancer. Proc. Natl. Acad. Sci. USA.

[61] Bianconi F., Palumbo I., Spanu A., Nuvoli S., Fravolini M.L., Palumbo B. (2020). PET/CT Radiomics in Lung Cancer: An Overview. Appl. Sci..

[62] Fraioli F., Lyasheva M., Porter J.C., Bomanji J., Shortman R.I., Endozo R., Wan S., Bertoletti L., Machado M., Ganeshan B. (2019). Synergistic Application of Pulmonary F-FDG PET/HRCT and Computer-Based CT Analysis with Conventional Severity Measures to Refine Current Risk Stratification in Idiopathic Pulmonary Fibrosis (IPF). Eur. J. Nucl. Med. Mol. Imaging.

