# Supplementary material for: A Low-Dose CT-Based Radiomic Model to Improve Characterization and Screening Recall Intervals of Indeterminate Prevalent Pulmonary Nodules
Source: Diagnostics (Basel). 2021 Sep 3;11(9):1610. doi: 10.3390/diagnostics11091610 (PMC8471292; doi:10.3390/diagnostics11091610)
Supplement: Supplementary file 1 [file diagnostics-11-01610-s001.zip › diagnostics-1343701-supplementary.pdf]

A Low-dose CT-based Radiomic Model to Improve Characterization and Screening Recall Intervals of Indeterminate Prevalent Pulmonary Nodules

Supplementary Materials

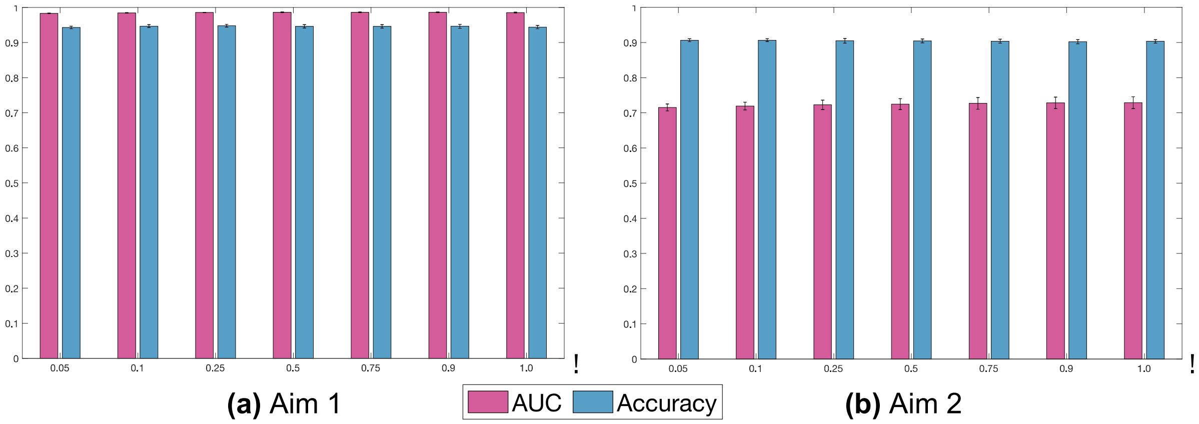

Figure S1: Hyper-parameter optimisation of the radiomic models in terms of the mixing parameter α (Elastic Net ℓ_1/ℓ_2 regularization parameter): **(a)** Aim 1; **(b)** Aim 2. The Elastic Net models were trained in 5-fold nested CV and the process was repeated 30 times. The considered evaluation metrics were AUC and accuracy.

(a)

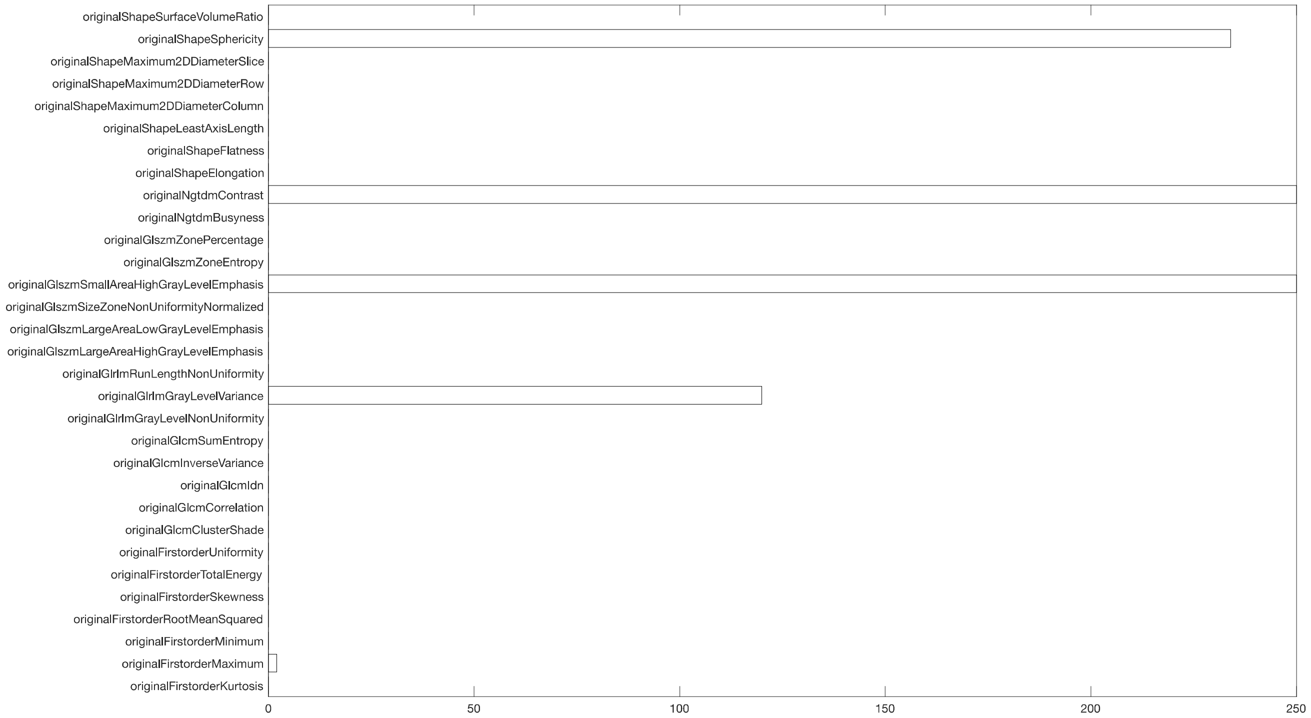

(b)

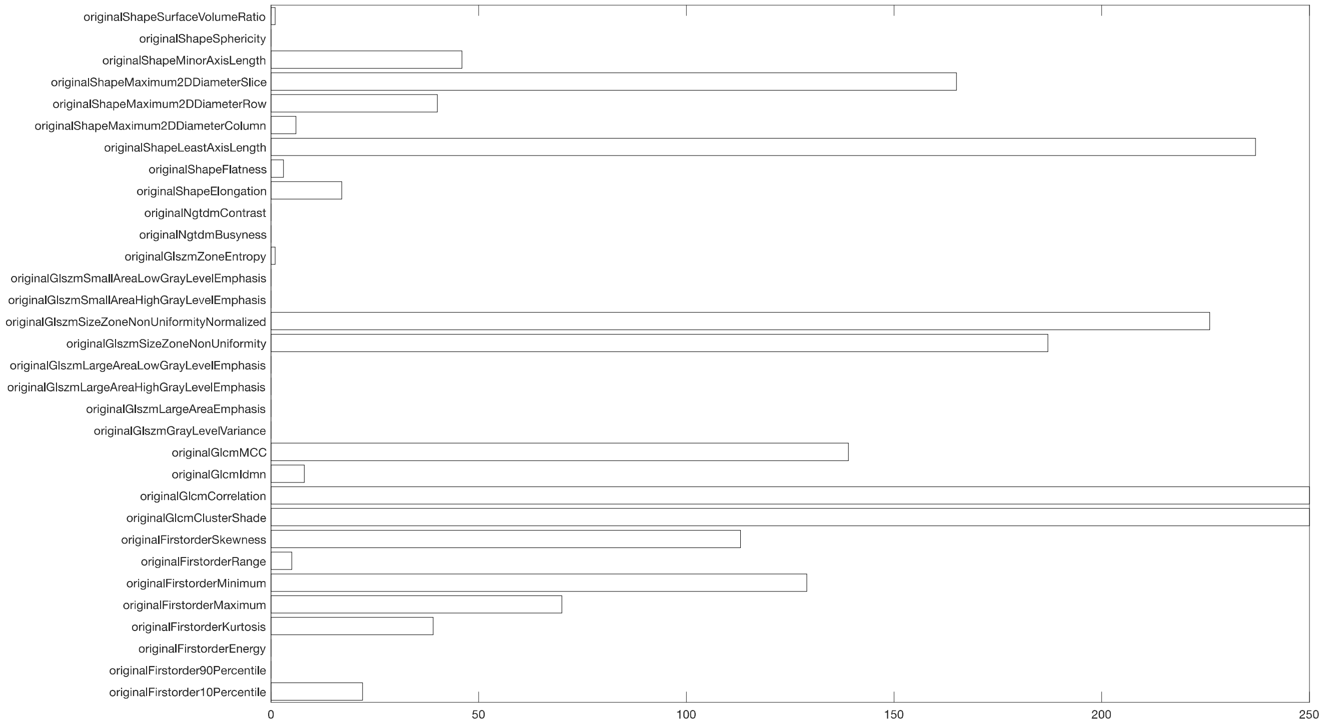

Figure S2: Most frequently selected radiomic feature analysis of the Elastic Net models considering the features selected after 50 repetitions on the nested 5-fold CV on the discovery cohort for: **(a)** Aim 1; **(b)** Aim 2. A total of 250 models were trained for each task.

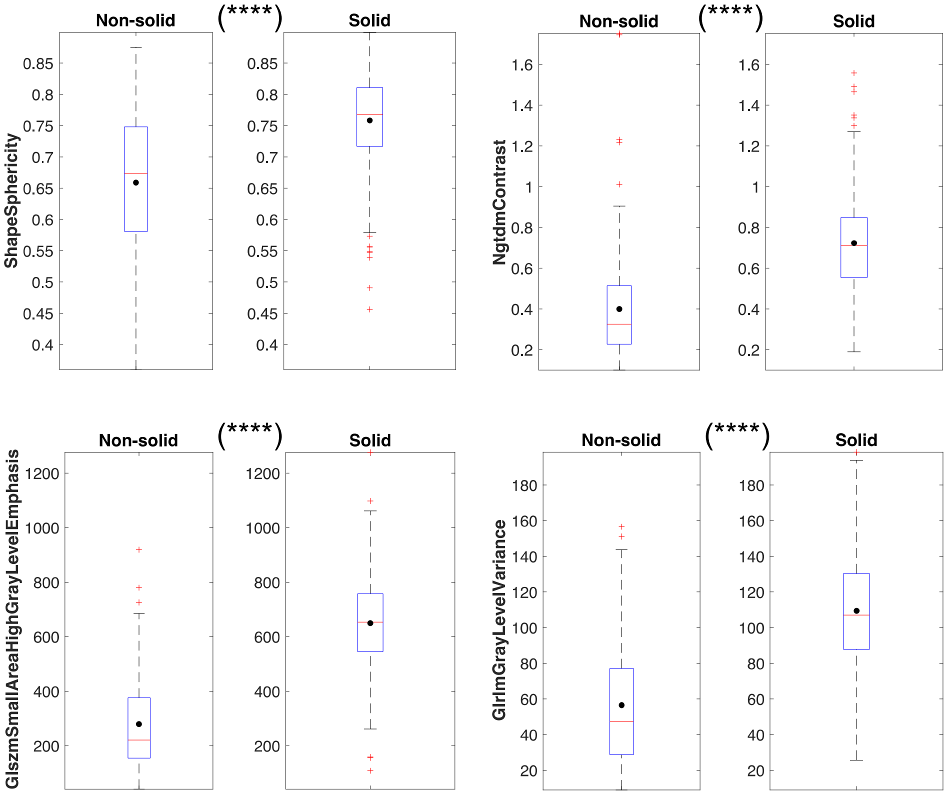

Figure S3: Boxplots of the four most frequently selected radiomic features for Aim 1 solid *vs.* sub-solid nodule classification on the discovery dataset. For each feature, a non-parametric Wilcoxon sum test (Mann-Withney U test) was performed by sub-dividing the samples into the two classes (significance level set to 0.05). The *p*-values were adjusted using the Bonferroni-Holm method for multiple comparison tests. Notation: * $p<0.05$. ** $p<0.01$, *** $p<0.001$, **** $p<0.0001$.

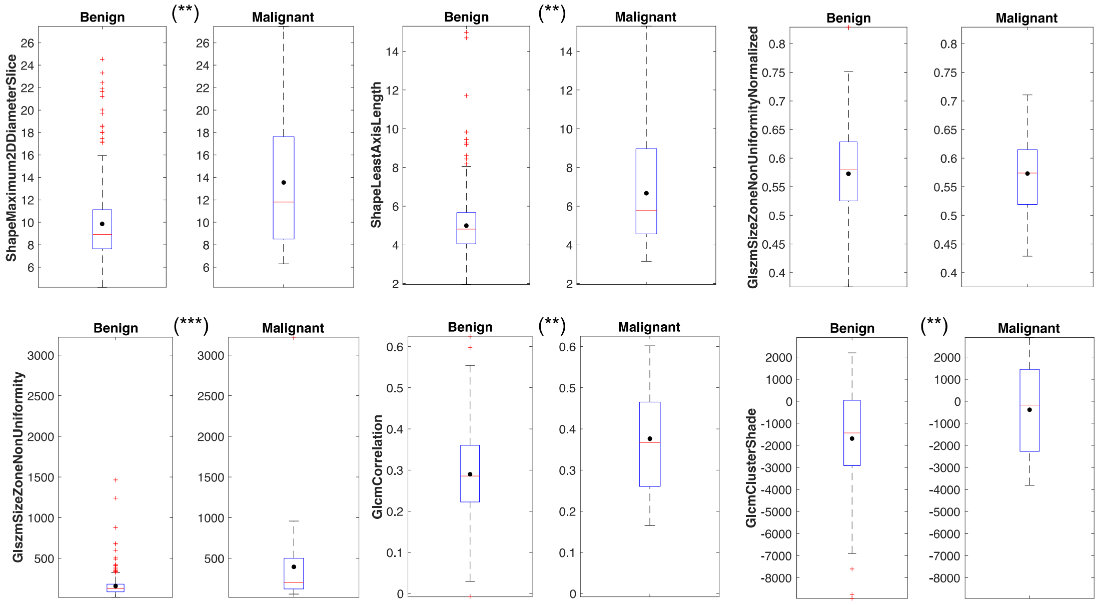

Figure S4: Boxplots of the six most frequently selected radiomic features for Aim 2 malignant *vs.* benign primary nodule classification on the discovery dataset. For each feature, a non-parametric Wilcoxon sum test (Mann-Withney U test) was performed by sub-dividing the samples into the two classes (significance level set to 0.05). The *p*-values were adjusted using the Bonferroni-Holm method for multiple comparison tests. Notation: * $p<0.05$. ** $p<0.01$, *** $p<0.001$, **** $p<0.0001$.

PCA and t-SNE results

Figure S5 shows the dimensionality reduction results for both methods on the discovery set of primary malignant nodules (Aim 1) for all the 107 radiomic features, as well as for the most frequently selected ones. While PCA is useful for plotting samples in a space with reduced dimensionality, t-SNE is useful for understanding the data distribution in a multidimensional manifold. The PCA showed that there is a very strong linear component (i.e., the first two main components have about 99.7% of explained variance); in both sub-figures, with greater detail in the case of the figure relating to the PCA with the relevant features, it is worth noting that there is an outlier in the original samples without which the distribution would appear totally different. In the case of the t-SNE plots, the samples concerning the most relevant features cover a higher portion of the space compared to the samples when all features were considered. The distribution of the synthetic samples is generated within the "convex hull" defined by the original samples, and this is due to the way the new synthetic samples were generated by the PCA.

Over-sampling distributions (Borderline-SMOTE)

Figure S6 shows the distributions of the synthetic samples generated by Borderline-SMOTE compared against the actual minority class samples (i.e., malignant primary nodules) included in the discovery set.

The synthetic distributions appear fairly overlapping with the original ones, and thus the synthetic data can be considered reliable and correctly generated for our purposes. In particular, the χ^2 test was used to assess the frequency distribution overlap. The synthetic distributions for GLCM Correlation and GLSZM Size Zone Non-Uniformity Normalized appear to be the most similar to the original dataset. Therefore, we can argue that the distributions are overlapping both qualitatively and quantitatively (considering the χ^2 test), thus justifying their use in the experiments employing minority class over-sampled samples. **Tables S3 and S4** present the performance achieved by the classifier for Aim 2 on the blinded test set, by using the investigated minority over-sampling and majority class under-sampling configurations, respectively.

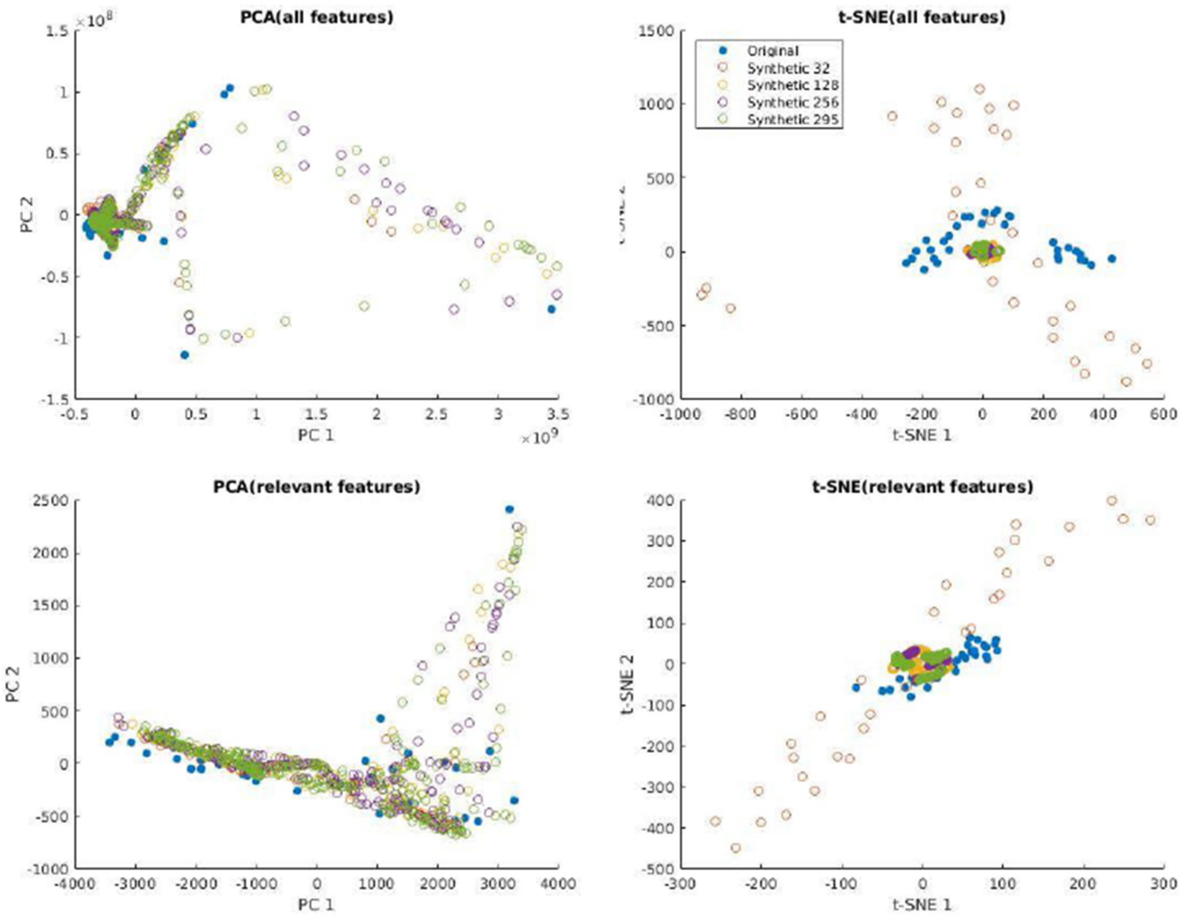

Figure S5. Dimensionality reduction to assess the data distribution for the synthetic samples generated by Borderline-SMOTE on the discovery set of primary malignant nodules (Aim 1) for all the 107 radiomic features, as well as for the most frequently selected ones.

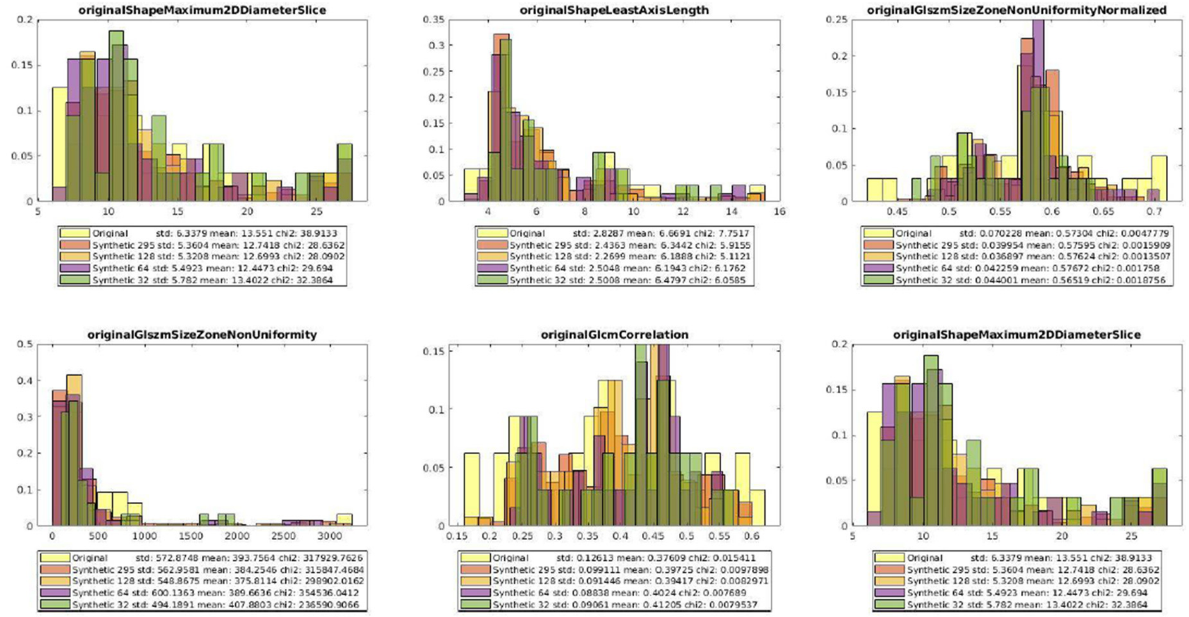

Figure S6. Distributions of the synthetic samples generated by Borderline-SMOTE compared against the actual minority class samples (i.e. malignant primary nodules) included in the discovery set. A plot for each of the most relevant features is shown.

Table S1. Results for the benign *vs.* malignant primary nodule classification (Aim 2) achieved by the nested 5-fold CV on the discovery set for each minority class over-sampling method under consideration. The metrics are expressed as average ± standard deviation. The rows in boldface denote the best performing configurations.

# Samples	AUC	Accuracy	PPV	NPV	Sensitivity	Specificity
**SMOTE**						
32	0.7103±0.0067	0.8331±0.0065	0.6081±0.0612	0.8551±0.0049	0.2502±0.0324	0.9601±0.0094
64	0.7298±0.0074	0.7963±0.0074	0.6595±0.0270	0.8206±0.0067	0.3710±0.0296	0.9352±0.0081
128	0.7397±0.0045	0.7374±0.0073	0.7260±0.0284	0.7469±0.0053	0.4341±0.0236	0.9025±0.0196
192	0.7473±0.0049	0.7103±0.0068	0.7225±0.0193	0.7126±0.0106	0.5545±0.0323	0.8290±0.0219
256	0.7391±0.0044	0.6882±0.0079	0.7309±0.0127	0.6642±0.0091	0.59241±0.0221	0.7821±0.0181
295	0.7438±0.0044	0.6907±0.0072	0.7467±0.0107	0.6489±0.0076	0.6263±0.0138	0.7624±0.0144
**Borderline-SMOTE**						
32	0.7682±0.0084	0.8258±0.0067	0.5684±0.0610	0.8572±0.0069	0.2920±0.0430	0.9456±0.0091
64	0.7876±0.0043	0.8090±0.0084	0.6691±0.0325	0.8388±0.0083	0.4316±0.0381	0.9283±0.0117
**128**	**0.8259±0.0035**	**0.7935±0.0069**	**0.7413±0.0126**	**0.8196±0.0076**	**0.6436±0.0206**	**0.8751±0.0089**
192	0.7952±0.0024	0.7827±0.0055	0.8062±0.0101	0.7724±0.0047	0.6574±0.0093	0.8782±0.0090
**256**	**0.8173±0.0031**	**0.7661±0.0055**	**0.8297±0.0091**	**0.7256±0.0041**	**0.6699±0.0063**	**0.8617±0.0089**
295	0.8021±0.0033	0.7485±0.0052	0.8396±0.0094	0.6882±0.0043	0.6477±0.0064	0.8605±0.0104
**Safe-Level-SMOTE**						
32	0.7156±0.0087	0.8156±0.0080	0.4906±0.0965	0.8373±0.0049	0.1502±0.0357	0.9628±0.0138
64	0.7242±0.0063	0.7781±0.0082	0.5713±0.0294	0.8274±0.0099	0.4071±0.0473	0.8968±0.0129
128	0.7091±0.0052	0.7267±0.0067	0.6674±0.0153	0.7484±0.0061	0.4608±0.0187	0.8723±0.0108
192	0.7507±0.0044	0.7226±0.0074	0.7334±0.0182	0.7218±0.0065	0.5703±0.0177	0.8382±0.0186
256	0.7721±0.0040	0.7305±0.0065	0.7627±0.0090	0.7104±0.0094	0.6652±0.0195	0.7945±0.0125
295	0.7764±0.0040	0.7334±0.0051	0.7779±0.0081	0.6968±0.0059	0.6928±0.0111	0.7786±0.0112
**ADASYN**						
32	0.7341±0.0078	0.8405±0.0053	0.6596±0.0736	0.8565±0.0039	0.2521±0.0252	0.9685±0.0069
64	0.7337±0.0059	0.7905±0.0078	0.6819±0.0334	0.8070±0.0055	0.3016±0.0255	0.9502±0.0090
128	0.7494±0.0051	0.7271±0.0076	0.6887±0.0231	0.7474±0.0076	0.4601±0.0248	0.8752±0.0155
192	0.7479±0.0056	0.7185±0.0077	0.7331±0.0130	0.7172±0.0076	0.5579±0.0166	0.8409±0.0143
256	0.7507±0.0051	0.7014±0.0087	0.7607±0.0159	0.6704±0.0060	0.5854±0.0117	0.8149±0.0186
295	0.7427±0.0051	0.6931±0.0080	0.7854±0.0126	0.6401±0.0068	0.5804±0.0098	0.8186±0.0139

Table S2. Results for the benign vs. malignant primary nodule classification (Aim 2) achieved by the nested 5-fold CV on the discovery set for each majority class under-sampling method under consideration. The metrics are expressed as average ± standard deviation.

# Samples	AUC	Accuracy	PPV	NPV	Sensitivity	Specificity
32	0.7730±0.0249	0.6316±0.0311	0.6344±0.0641	0.6695±0.0717	0.6504±0.0796	0.6182±0.0686
64	0.7679±0.0194	0.7649±0.0184	0.7704±0.0820	0.7765±0.0130	0.4563±0.0394	0.9186±0.0332
96	0.7464±0.0149	0.7910±0.0111	0.6825±0.0905	0.8125±0.0094	0.3372±0.0454	0.9418±0.0176
128	0.7387±0.0169	0.8081±0.0124	0.5557±0.0128	0.8336±0.0086	0.2337±0.0509	0.9519±0.0176
256	0.7141±0.0143	0.8874±0.0064	0.4464±0.0150	0.9041±0.0041	0.1670±0.0414	0.9774±0.0067

Table S3. Results for the benign *vs.* malignant primary nodule classification (Aim 2) achieved by the fitted models (in nested 5-fold CV) on the blinded test set for each minority class over-sampling method under consideration. The metrics are expressed as average ± standard deviation. The rows in boldface denote the best performing configurations.

# Samples	AUC	Accuracy	PPV	NPV	Sensitivity	Specificity
**SMOTE**						
32	0.6110±0.0147	0.8587±0.0109	0.3802±0.0666	0.8923±0.0044	0.1938±0.0388	0.9536±0.0130
64	0.6107±0.0176	0.8256±0.0190	0.2759±0.0558	0.8918±0.0027	0.2262±0.0185	0.9112±0.0223
128	0.5922±0.0120	0.7038±0.0282	0.1834±0.0153	0.8969±0.0067	0.3969±0.0666	0.7477±0.0394
192	0.5670±0.0099	0.6056±0.0121	0.1501±0.0047	0.8905±0.0021	0.4615±4.485e-16	0.6262±0.0138
256	0.5487±0.0117	0.5562±0.0213	0.1378±0.0081	0.8849±0.0063	0.4846±0.0356	0.5664±0.0265
295	0.5288±0.0141	0.5154±0.0196	0.1301±0.0066	0.8799±0.0063	0.5062±0.0384	0.5167±0.0256
**Borderline-SMOTE**						
32	0.6015±0.0124	0.8381±0.0185	0.3168±0.0674	0.8936±0.0023	0.2292±0.0109	0.9251±0.0214
64	0.5913±0.0161	0.8046±0.0180	0.2290±0.0276	0.8897±0.0023	0.2308±2.243e-16	0.8866±0.0205
**128**	**0.5711±0.0089**	**0.6965±0.0177**	**0.1633±0.0111**	**0.8889±0.0042**	**0.3462±0.0389**	**0.7466±0.0239**
192	0.5740±0.0079	0.6431±0.0084	0.1650±0.0063	0.8962±0.0031	0.4569±0.0185	0.6697±0.0098
**256**	**0.5559±0.0067**	**0.6031±0.0099**	**0.1491±0.0038**	**0.8901±0.0018**	**0.4615±4.486e-16**	**0.6233±0.0113**
295	0.5475±0.0087	0.5838±0.0093	0.1420±0.0033	0.8866±0.0018	0.4615±4.486e-16	0.6013±0.0107
**Safe-Level-SMOTE**						
32	0.6217±0.0148	0.8725±0.0058	0.4794±0.0580	0.8954±0.0039	0.2092±0.0349	0.9673±0.0065
64	0.5926±0.0158	0.8051±0.0198	0.2321±0.0414	0.8897±0.0025	0.2308±2.243e-16	0.8870±0.0226
128	0.5838±0.0123	0.7023±0.0175	0.1698±0.0156	0.8909±0.0056	0.3554±0.0437	0.7519±0.0224
192	0.5659±0.0147	0.6171±0.0148	0.1525±0.0082	0.8912±0.0042	0.4523±0.0253	0.6407±0.0177
256	0.5560±0.0121	0.5631±0.0216	0.1536±0.0132	0.8987±0.0112	0.5538±0.0602	0.5644±0.0269
295	0.5633±0.0096	0.5104±0.0223	0.1486±0.0064	0.8999±0.0047	0.6154±4.486e-16	0.4954±0.0255
**ADASYN**						
32	0.6089±0.0129	0.8602±0.0103	0.3830±0.0623	0.8908±0.0037	0.1769±0.0356	0.9578±0.0138
64	0.5977±0.0166	0.8154±0.0207	0.2481±0.0530	0.8899±0.0033	0.22±0.0270	0.9004±0.0249
128	0.5856±0.0108	0.7181±0.0197	0.1864±0.0157	0.8952±0.0042	0.3708±0.0299	0.7677±0.0238
192	0.5610±0.0147	0.6152±0.0274	0.1385±0.0117	0.8820±0.0055	0.3954±0.0270	0.6466±0.0326
256	0.5525±0.0107	0.5787±0.0163	0.1483±0.0081	0.8921±0.0063	0.5±0.0389	0.5899±0.0212
295	0.5531±0.0106	0.5710±0.0147	0.1421±0.0089	0.8877±0.0068	0.4831±0.0382	0.5835±0.0182

Table S4. Results for the benign *vs*. malignant primary nodule classification (Aim 2) achieved by the fitted models (in nested 5-fold CV) on the blinded test set for each minority class under-sampling method under consideration. The metrics are expressed as average ± standard deviation.

# **Samples**	**AUC**	**Accuracy**	**PPV**	**NPV**	**Sensitivity**	**Specificity**
32	0.5497±0.0273	0.4521±0.0930	0.1301±0.0170	0.8777±0.0224	0.5831±0.0996	0.4334±0.1163
64	0.5806±0.0160	0.7662±0.0196	0.2129±0.0199	0.8950±0.0034	0.3184±0.0311	0.8301±0.0250
96	0.5822±0.0169	0.7798±0.0222	0.1998±0.0368	0.8885±0.0060	0.2477±0.0545	0.8558±0.0290
128	0.5703±0.0165	0.8573±0.0195	0.3878±0.0969	0.8909±0.0043	0.1831±0.0408	0.9536±0.0245
256	0.6254±0.0082	0.8742±0.0026	0.4907±0.0330	0.8896±0.0016	0.1508±0.0152	0.9776±0.0031

Table S5. Radiomic features extracted from the VOIs in this study. All radiomic features were extracted using PyRadiomics and the radiomic feature formulation can be found on the online PyRadiomics documentation (https://pyradiomics.readthedocs.io/en/latest/)

#	**Radiomic feature**
First-order	
1	10th Percentile
2	90th Percentile
3	Energy
4	Entropy
5	Interquartile Range
6	Kurtosis
7	Maximum
8	Mean Absolute Deviation
9	Mean
10	Median
11	Minimum
12	Range
13	Robust Mean Absolute Deviation
14	Root Mean Squared
15	Skewness
16	Total Energy
17	Uniformity

18	Variance
Shape-based (3D)	
19	Mesh Volume
20	Voxel Volume
21	Surface Area
22	Surface Area to Volume ratio
23	Sphericity
24	Maximum 3D diameter
25	Maximum 2D diameter (slice)
26	Maximum 2D diameter (column)
27	Maximum 2D diameter (row)
28	Major Axis Length
29	Minor Axis Length
30	Least Axis Length
31	Elongation
32	Flatness
Gray Level Co-occurrence Matrix (GLCM)	
33	Autocorrelation
34	Cluster Prominence
35	Cluster Shade
36	Cluster Tendency
37	Contrast

38	Correlation
39	Difference Average
40	Difference Entropy
41	Difference Variance
42	ID: Inverse Difference
43	IDM: Inverse Difference Moment
44	IDMN: Inverse Difference Moment Normalized
45	IDN: Inverse Difference Normalized
46	IMC 1: Informational Measure of Correlation 1
47	IMC 2: Informational Measure of Correlation 2
48	Inverse Variance
49	Joint Average
50	Joint Energy
51	Joint Entropy
52	MCC: Maximal Correlation Coefficient
53	Maximum Probability
54	Sum Average
55	Sum Entropy
56	Sum Squares
Gray Level Dependence Matrix (GLDM)	
57	Dependence Entropy
58	Dependence NonUniformity

59	Dependence NonUniformity Normalised
60	Dependence Variance
61	Gray Level NonUniformity
62	Gray Level Variance
63	High Gray Level Emphasis
64	Large Dependence Emphasis
65	Large Dependence High Gray Level Emphasis
66	Large Dependence Low Gray Level Emphasis
67	Low Gray Level Emphasis
68	Small Dependence Emphasis
69	Small Dependence High Gray Level Emphasis
70	Small Dependence Low Gray Level Emphasis
Gray Level Run Length Matrix (GLRLM)	
71	Gray Level NonUniformity
72	Gray Level NonUniformity Normalised
73	Gray Level Variance
74	High Gray Level Run Emphasis
75	Long Run Emphasis
76	Long Run High Gray Level Emphasis
77	Long Run Low Gray Level Emphasis
78	Low Gray Level Run Emphasis
79	Run Entropy

80	Run Length NonUniformity
81	Run Length NonUniformity Normalised
82	Run Percentage
83	Run Variance
84	Short Run Emphasis
85	Short Run High Gray Level Emphasis
86	Short Run Low Gray Level Emphasis
Gray Level Size Zone Matrix (GLSZM)	
87	Gray Level NonUniformity
88	Gray Level NonUniformity Normalised
89	Gray Level Variance
90	High Gray Level Zone Emphasis
91	Large Area Emphasis
92	Large Area High Gray Level Emphasis
93	Large Area Low Gray Level Emphasis
94	Low Gray Level Zone Emphasis
95	Size Zone NonUniformity
96	Size Zone NonUniformity Normalised
97	Small Area Emphasis
98	Small Area High Gray Level Emphasis
99	Small Area Low Gray Level Emphasis
100	Zone Entropy

101	Zone Percentage
102	Zone Variance
Neighboring Gray-Tone Difference Matrix (NGTDM)	
103	Busyness
104	Coarseness
105	Complexity
106	Contrast
107	Strength

Table S6. Characteristics of the proposed radiomics study according to the reporting guidelines provided by the Image Biomarker Standardization Initiative (IBSI) [Chapter 4].

Topic	Item	Description
Patient		
Region of interest	1	Lungs, pulmonary nodules
Patient preparation	2a	Subjects were only instructed to take a deep breath in during images acquisition, according to the CT scanner instructions
	2b	None
	2c	None
Contrast agent	4a	None
Acquisition		
Acquisition protocol	6	A low dose CT protocol was used for all subjects included. Detailed information is reported in Section 2.1.1 of the main text.
Scanner type	7	Second-generation dual-source CT scanner (Somatom Definition Flash; Siemens Medical Solutions; Forchheim, Germany).
Imaging modality	8	Computed Tomography
Static/dynamic scans	9a	Static

Scanner calibration	10	Routine scanner calibration was performed at the beginning of each session
Patient instructions	11	Subjects lying in the supine position were instructed to hold a deep breath in during images acquisition
Anatomical motion correction	12	None
Scan rotation time	13	0.5 sec
Tube voltage	14	120 kVp
Tube current	15	30 mAs
Reconstruction		
In-plane resolution	28	Matrix size of 512x512
Image slice thickness	29	1 mm
Image slice spacing	30	0.7 mm
Convolution kernel	31a	Medium-sharp kernel (B50f)
Image registration		
Registration method	37	Not applicable since only CT images were analyzed
Image processing - data conversion		
Other data conversions	40	None
Image processing – post-acquisition processing		
Anti-aliasing	41	None
Noise suppression	42	None
Other post-acquisition processing methods	47	None
Segmentation		
Segmentation method	48a	Semi-automated with manual refinements
	48b	Segmentations were performed by a radiologist with one year of expertise in thoracic imaging
	48c	Pulmonary nodules were semi-automatically delineated every two slices through manually drawn regions of interest (ROIs) and the remaining slices were interpolated accordingly. A dedicated algorithm tool was then used to calculate a volume of interest (VOI), including the whole lesions. In case of inaccurate segmentation, the operator was allowed to modify VOI

		boundaries.
Image processing – image interpolation		
Interpolation method	50a	None
	50b	None
	50c	None
	50d	None
Voxel dimensions	51	None
Intensity rounding	52	None
Image processing – ROI interpolation		
Interpolation method	53	Linear
Partially masked voxels	54	Inaccurate interpolation results were modified manually by the operator
Image processing – re-segmentation		
Re-segmentation methods	55	Not applied
Image processing - discretisation		
Discretization method	56a	Fixed bin width
	56b	Bin width = 25 (default in PyRadiomics)
	56c	No re-segmentation range was used
Image processing – image transformation		
Image filter	57	Not currently used
Image biomarker computation		
Biomarker set	58	107 radiomic features, extracted using a segmentation software built-in function, named SlicerRadiomics, which integrates the tool PyRadiomics.<br>Six classes of features were obtained: (1) first-order intensity histogram statistics, (2) Gray Level Co-occurrence Matrix features (GLCM), (3) Gray Level Run Length Matrix (GLRLM), (4) Gray Level Size Zone Matrix (GLSZM), (5) Gray Level Dependence Matrix (GLDM), and (6) Neighboring Gray Tone Difference Matrix (NGTDM).
IBSI compliance	59	The software used that is aimed at measuring standardized radiomic features is fully compliant with the IBSI standard.

Robustness	60	Not currently present
Software availability	61	SlicerRadiomics + PyRadiomics v2.2.0 (*via* 3D Slicer 4.10.0)
Image biomarker computation – texture parameters		
	62–71	Default settings of PyRadiomics (for full reproducibility)
Machine learning and radiomics analysis		
Diagnostic and prognostic modelling	72	The TRIPOD Checklist for Prediction Model Development and Validation was used
Comparison with known factors	73	The radiomics model was compared to the characterization and screening LDCT recall intervals of subjects with indeterminate prevalent pulmonary nodules
Multicollinearity	74	Non-redundant feature analysis based on the Spearman correlation coefficient
Model availability	75	Available upon reasonable request
Data availability	76	Data are not publicly available due to restrictions for ethical reasons.
